# Eclampsia

**DOI:** 10.21980/J8PS8R

**Published:** 2021-07-15

**Authors:** Thomas J Yang, Rohit B Sangal, Lauren W Conlon

**Affiliations:** * Yale University, Department of Emergency Medicine, New Haven, CT; ^ University of Pennsylvania, Department of Emergency Medicine, Philadelphia, PA

## Abstract

**Audience:**

Emergency medicine residents.

**Introduction:**

Eclampsia is the development of a generalized seizure in pregnant patients with hypertension of pregnancy.^1^ Eclampsia exists on the spectrum of hypertension-related disorders in pregnancy, occurs in 1 out of 1,000–10,000 deliveries,^1^–^3^and is associated with significant maternal and fetal morbidity and mortality.^4^ Given the emergent nature of eclampsia and the benefit of rapid treatment, emergency medicine (EM) physicians need to quickly recognize and treat this rare pathology. Although residents have three to four years before becoming an attending, not all emergent pathologies may present clinically during their training. It is important to simulate rare, treatable conditions such as eclampsia to give learners exposure confidence in managing this disease.

**Educational Objectives:**

By the end of this simulation session, learners will be able to:

1. Demonstrate care of a gravid patient with altered mental status
2. Demonstrate care of a gravid patient with seizures
3. Recognize care involved in assessment of fetal status
4. Execute appropriate subspecialty consultation
5. Recognize the clinical signs and symptoms of eclampsia
6. Distinguish different treatment options for eclampsia
7. Identify magnesium toxicity and reversal agent
8. Differentiate the spectrum of preeclampsia

**Educational Methods:**

As an educational strategy, simulation allows learners to partake in experiential learning. By creating a safe and supportive learning environment, simulation allows learners to facilitate deliberate practice and transfer learning in debriefing sessions. High-fidelity sessions involve software and technology to mimic realistic patient environments, which also activate learners’ affective states to aid in decision-making abilities in complex medical cases.

This session was conducted using a high-fidelity mannequin, SimMom (Laerdal), and a controlling Laerdal LLEAP Software. Faculty-led debriefing followed the simulation case and included discussion regarding presentation, spectrum, and management of the obstetrical emergency.^5^

**Research Methods:**

Resident participants completed an evaluation form consisting of questions on a 5-point Likert scale assessing the realism and usefulness of the simulation.

**Results:**

All 18 residents who participated in the simulation completed an evaluation form, and all agreed or strongly agreed the case was realistic and useful.

**Discussion:**

Incorporating high-stakes, low-frequency presentations through simulation can be readily applied in residency education and well-received by residents. Increasing the difficulty through adjusting the clinical history and exam may challenge learners further.

**Topics:**

Medical simulation, eclampsia, pregnancy, obstetrics, emergency medicine.

## USER GUIDE

[Table t1-jetem-6-3-s33]

**Table t1-jetem-6-3-s33:** 

**List of Resources:**	
Abstract	33
User Guide	35
Instructor Materials	38
Operator Materials	48
Debriefing and Evaluation Pearls	52
Simulation Assessment	57

**Learner Audience:**

Medical students, emergency medicine interns, junior residents, senior residents

**Time Required for Implementation:**

Instructor Preparation: 20–30 minutes

Time for case: 15–20 minutes

Time for debriefing: 10–30 minutes

**Recommended Number of Learners per Instructor:** 3

**Topics:**

Medical simulation, eclampsia, pregnancy, obstetrics, emergency medicine.

**Objectives:**

By the end of this simulation session, the learner will be able to:

1. Demonstrate care of a gravid patient with altered mental status
2. Demonstrate care of a gravid patient with seizures
3. Recognize care involved in assessment of fetal status
4. Execute appropriate subspecialty consultation
5. Recognize the clinical signs and symptoms of eclampsia
6. Distinguish different treatment options for eclampsia
7. Identify magnesium toxicity and reversal agent
8. Differentiate the spectrum of preeclampsia

### Linked objectives and methods

Simulation can be used for the purpose of practicing clinical care for rare or high-risk pathology, among a variety of other reasons.^8^ ^9^ Simulation can help learners gain confidence and skills that help them perform in high acuity situations.^9^ This case is based on a pregnant patient who presents via ambulance with a clinical history and exam concerning for eclampsia. Learners need to begin management of a gravid patient with altered mental status and seizures (objective 1 and 2). They should perform an initial diagnostic workup for the suspected pathology, fetal monitoring, and consult the appropriate service (objectives 3 and 4). The consult will not be available immediately to allow the learner to continue working through the case. The learner needs to recognize the clinical signs and symptoms of eclampsia (objective 5). The learner should begin to treat the patient’s blood pressure (objective 6) and eventually assess the need for managing magnesium toxicity (objective 7). The simulation ends when the patient is admitted to the intensive care unit (ICU) or to the operating room (OR) for emergent delivery. The debriefing will review the objectives of the case, relevant clinical features of eclampsia, and a discussion of the spectrum of preeclampsia (Objectives 1–8).

### Recommended pre-reading for instructor

Recommended reading includes resources on eclampsia and emergency diagnosis and treatment.^6^ ^7^ This can be supplemented with current guidelines from the American College of Obstetricians and Gynecologists, references within this case, and any hospital protocols related to eclampsia. If available, such institutional protocols should be shown to learners for real-time reinforcement.

### Results and tips for successful implementation

This case was performed with a high fidelity simulator using SimMom (Laerdal). While this could be done with a standardized patient if a mannequin is unavailable, the prolonged seizure display may be challenging for a standardized patient. Standard emergency department supplies were available, including intravenous (IV) line supplies, IV fluids, code medications, defibrillator, and common ED medications. Imaging and laboratory data are available at learner request if appropriate for the case. Supply lists are provided within the case materials for set up.

This simulation was taught to 18 EM residents in a four-year residency program. Prior to beginning the simulation, learners were oriented to the simulation center and high-fidelity mannequins. Learners were divided into groups by residency leadership to distribute skill levels across teams. Given limited decision-makers in this case, we recommend groups of three. Of note, we assigned groups of four or five given the presence of medical student rotators and limited time availability of simulation equipment. The operator of the mannequin can be anyone with a script to the case but should have basic knowledge of how to communicate through the mannequin and change digital vital signs. Finally, an EM faculty member observer was present in the room to evaluate the team as well as troubleshoot any unexpected issues. The faculty member had a list of predetermined critical actions on which to evaluate the team and a debriefing guide that outlined the objectives of the case.

At the end of the debriefing, residents completed an evaluation form for the case. 18 out of 18 (100%) residents completed an evaluation. Six PGY-1, five PGY-2, two PGY-3, and five PGY-4s completed the surveys. Surveys were anonymous, and the University Institutional Research Board approved this study. All participants agreed (n =13) or strongly agreed (n=5) the simulation was realistic and useful. Selected comments are below:

Positive:

- “Seems very bread and butter, known problem, known solution and a good review of the standard medications” (PGY 1)
- “I liked the monitoring for med toxicity/effects” (PGY 2)
- “A rare case is always helpful to do especially without OB present” (PGY 3)
- “Was realistic and nice to review what we do not see every day” (PGY 4)

Some suggested comments for improvement include “having a family to talk to,” “access to ultrasound images,” and “making IV access difficult.” Of note, the structure of the university ED has a separate OB triage managed by the OBGYN department; pregnancy-related complaints above 15 weeks are not often seen in the university ED. This likely influenced resident positive reception of the case and comments related to not seeing such pathology. If senior residents desire increased difficulty, this case could be modified using a postpartum patient or a past medical history of diabetes or epilepsy to present alternative treatment considerations for seizures.

### Survey Instrument

[Fig f1-jetem-6-3-s33]

**Figure f1-jetem-6-3-s33:**
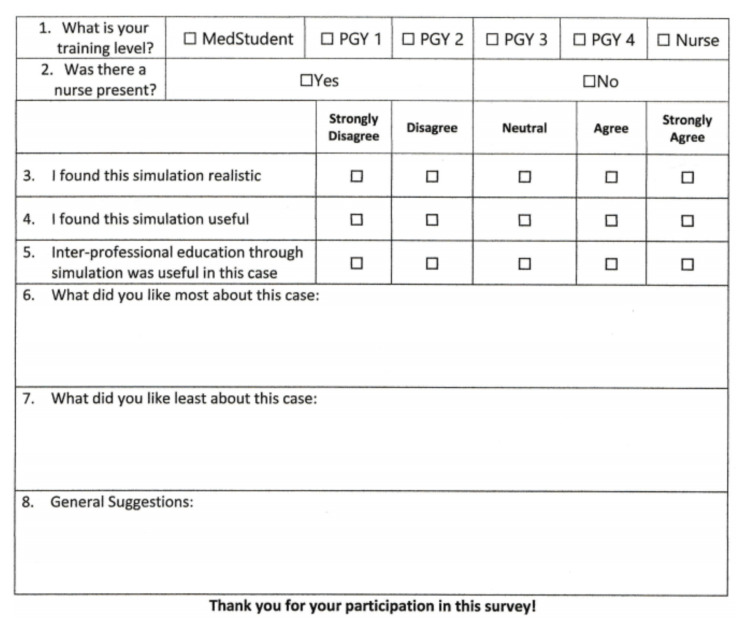


## Supplementary Information



## Figures and Tables

**Table t2-jetem-6-3-s33:** 

White blood count (WBC)	12.9 x1000/mm^3^
Hemoglobin (Hgb)	9.7 g/dL
Hematocrit (HCT)	24.6%
Platelet (Plt)	95 x1000/mm^3^
Diff	pending

**Table t3-jetem-6-3-s33:** 

Sodium	136 mEq/L
Chloride	108 mEq/L
Potassium	3.8 mEq/L
Bicarbonate (HCO_3_)	28 mEq/L
Blood Urea Nitrogen (BUN)	24 mg/dL
Creatine (Cr)	1.8 mg/dL
Glucose	98 mg/dL
Calcium	9.2 mg/dL
Total bilirubin	1.3 mg/dL
Direct bilirubin	0.8 mg/dL
Indirect bilirubin	0.3 mg/dL
Alkaline phosphatase	55 units/L
Aspartate aminotransferase (AST)	95 units/L
Alanine aminotransferase (ALT)	83 units/L
Albumin	2.8 g/dL

**Table t4-jetem-6-3-s33:** 

*Finger-stick blood glucose*	93 mg/dL

**Table t5-jetem-6-3-s33:** 

pH	7.36
PaCO2	61 mmHg
PaO2	45 mmHg
Bicarbonate (HCO_3_)	22 mEq/L

**Table t6-jetem-6-3-s33:** 

Color	yellow
Appearance	clear
Specific gravity	1.0
pH	7.0
Glucose	negative
Bilirubin	negative
Ketones	0
Protein	2+
Leukocyte esterase	negative
Nitrites	negative
White blood cells (WBC)	0 WBCs/high powered field (HPF)
Red blood cells (RBC)	0 RBCs/HPF
Squamous epithelial cells	0–5 cells/HPF

**Table t7-jetem-6-3-s33:** 

Amphetamine	negative
Barbiturate	negative
Benzodiazepine	negative
Cocaine	negative
Marijuana	negative
Methadone	negative
Methamphetamine	negative
Opiate	negative
Phencyclidine	negative
Tricyclic antidepressants	negative

**Table t8-jetem-6-3-s33:** 

*Urine Pregnancy*	positive

**Table t9-jetem-6-3-s33:** 

Prothrombin time (PT)	12.3 seconds
Partial thromboplastin time (PTT)	23.7 seconds
INR	1.1

**Figure f2-jetem-6-3-s33:**
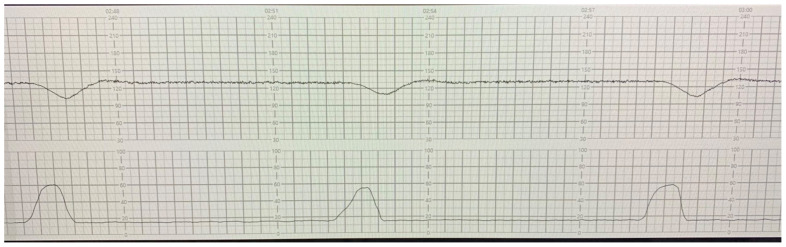


**Table t10-jetem-6-3-s33:** 

*Magnesium*	1.8 mEq/L

**Table t11-jetem-6-3-s33:** 

*Uric Acid*	pending
*LDH*	pending
*Haptaglobin*	pending
*Lactic Acid*	6.6 mmol/L
*Repeat Magnesium*	pending
*Repeat Lactic Acid*	1.8 mmol/L

**Figure f3-jetem-6-3-s33:**
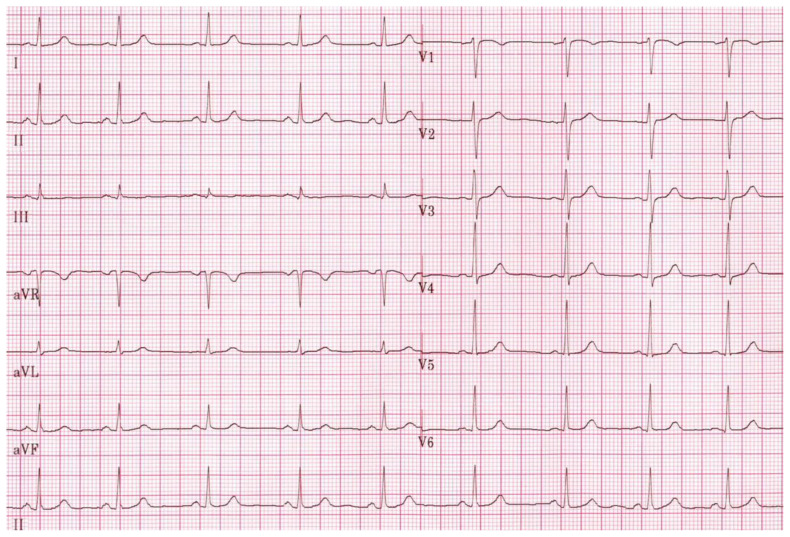


**Figure f4-jetem-6-3-s33:**
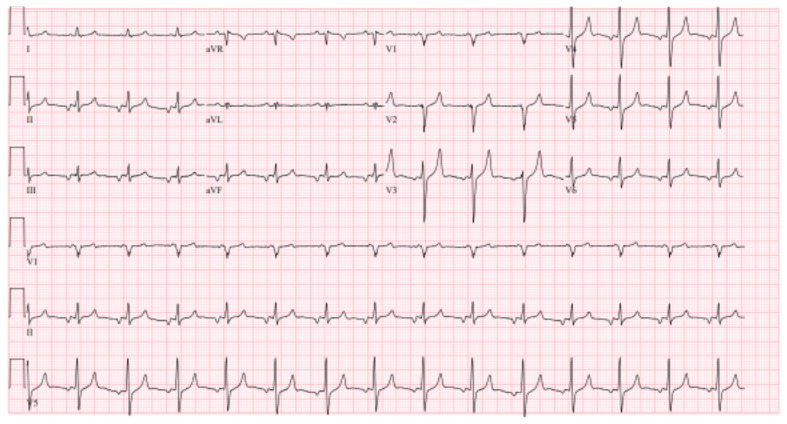


**Table t12-jetem-6-3-s33:** 

*Phosphorus*	2.6 mEq/L

**Table t13-jetem-6-3-s33:** SIMULATION EVENTS TABLE:

Minute (state)	Participant action/ trigger	Patient status (simulator response) & operator prompts	Monitor display (vital signs)

0:00 (Baseline)	Paramedic report.	Patient moaning, confused, states, “my head hurts,” but no other history (GCS 13). Primary Survey - Intact	T 99.0°F (both temporal or rectal) HR 118 BP 180/115 RR 24 O2 95% RA
	Place patient on the monitor		
	Learner obtains focused physical exam (primary exam)		

1:00–2:00	Give supplemental oxygen	SPO2 will improve if given oxygen	
	Obtain finger-stick blood glucose	If glucose not obtained, nursing can prompt later in case when patient begins to seize	T 99.0°F (both temporal or rectal) HR 110 BP 186/112 RR 22 O2 98% on 2LNC
	Peripheral IV, obtain basic history and undress patients/secondary exam	Obtain labs (CMP, CBC, coags, HCG, LFTs)	

2:00–4:00	Recognizes patient is pregnant - request Fetal Heart Rate (FHR) monitor and OB consult	Fetal heart tracing with HR in 130s, moderate variability, occasional late decelerations. OB resident in OR and is delayed. Patient able to provide limited history (if asked)	T 99.0°F HR 109 BP 205/112 RR 20 O2 94% on 2LNC (100% if started on NRB)
	Recognizes patient pregnant - request OB consult only	OB resident in OR and is delayed. Patient still postictal	
	Does not recognize patient pregnant	Patient still postictal	

4:00–6:00	Decide to treat HTN with labetalol (10–20mg IV) or hydralazine (5–10mg IV, max bolus 20mg IV)	BP improving initially	HR 100 BP 190/98 Remaining vitals unchanged
	Treat with nitroprusside	Fetal cyanide poisoning, fetal distress with repetitive late decelerations, HR 90s on FHR monitor	HR 100 BP 190/98 Remaining vitals unchanged
	Inappropriate dosing or forget to redose	BP unresponsive	Vitals unchanged
	No treatment of BP	BP slowly increases, patient with 30sec self-limited seizure	T 99.0°F HR 115 BP 220/120 RR 20 O2 94% on 2LNC (100% if started on NRB)

6:00–8:00	Repeat dose of labetalol (20–80mg IV to max of total 300mg IV) or hydralazine (5–10mg IV to max dose 30mg or switch to new agent)	BP continue to improve	T 99.0°F HR 90 BP 165/90 RR 20 O2 94% on 2LNC (100% if started on NRB)
	Forget to redose	BP slowly increases	T 99.0°F HR 115 BP 220/120 RR 20 O2 94% on 2LNC(100% if started on NRB)

8:00–9:00	Recognizes eclampsia and decides to treat with magnesium sulfate (4–6g over 20min, then 1–2g/hr infusion) but does not do other anti-HTN treatments	Patient with some improvement in confusion and able to provide limited history and mild improvement in BP	T 99.0°F HR 115 BP 195/100 RR 20 O2 94% on 2LNC (100% if started on NRB)
	Treats BP but does not recognize eclampsia as cause of seizures	Seizures begin despite BP improvement and SPO2 drops requiring intubation	T 99.0°F HR 118 BP 165/90 RR 10 O2 84%
	Treats both eclampsia and hypertension	No more seizure activity, BP as above and patient stabilizes	T 99.0°F HR 102 BP 165/90 RR 18 O2 100% on NRB

9:00–10:00	Does not recognize patient is pregnant or no treatment of HTN/seizure and instead gets head CT	Patient has generalized tonic-clonic seizure in CT	T 99.0°F HR 118 BP 165/90 RR 10 O2 84% on NRB
	If only benzodiazepines are given for seizures	Seizures briefly improve but then continue. Respiratory rate drops with benzodiazepines, eventually hypoxia requiring intubation	T 99.0°F HR 108 BP 190/105 RR 10
		If FHR is on, HR to 130s with late decelerations with repeated benzodiazepines	O2 84% on NRB

10:00–11:00	If appropriately managed thus far (magnesium and anti-HTN medications)	Seizures stop and patient waking up but feels muscle aches, diffuse weakness, nausea and drowsiness. Patient able to provide full history. Loss of deep tendon reflexes	T 99.0°F HR 105 BP 165/90 RR 18 O2 100% on NRB

11:00–13:00	Recognize magnesium toxicity and give calcium gluconate 1g IV	Patient feels better, symptoms resolve. No more muscle weakness, nausea or somnolence. Patient able to provide full history, interactive with staff. Deep tendon reflexes now present (if checked)	T 99.0°F HR 105 BP 165/90 RR 18 O2 100% on NRB
	Recognize magnesium toxicity and call renal for dialysis	Renal not in house and will see patient in the morning	
	Does not recognize magnesium toxicity	Patient starts to become bradycardic and hypotensive. Then, OBGYN team rushes in and take the patient to the OR and END the case.	T 99.0°F HR 40 BP 90/40 RR 18 O2 100% on NRB

13:00–15:00 (Case complete)	If OB was never called	Continues to have seizures refractory to treatment, intubation secondary to mental status and hypoxia. *OB team then shows up and calls the OR to make arrangements for the patient*	T 99.0°F HR 125 BP 190/105 RR 18 O2 84% on NRB

(Case Complete)	Disposition to OR and ICU	OB can arrive in ED and prompt learners. Also, family asks for the update and the plan for the patient care. This ends the scenario	

**Table t14-jetem-6-3-s33:** 

**Critical Actions:** Place IV, obtain vitals and a finger-stick glucose Perform primary survey (airway, breathing, circulation) Obtain a basic history of symptoms Perform physical exam and recognize pregnancy Verbalize “eclampsia” and call obstetrics consult early Emergent blood pressure management (labetalol or hydralazine) Treat seizure with magnesium Recognize magnesium toxicity Provide antidote for magnesium toxicity Clearly communicate with consultants and nurses with the management goals and disposition	0:00

**Table t15-jetem-6-3-s33:** 

	Milestone	Did not achieve level 1	Level 1	Level 2	Level 3
1	**Emergency Stabilization (PC1)**	□ Did not achieve Level 1	□ Recognizes abnormal vital signs	□ Recognizes an unstable patient, requiring intervention Performs primary assessment Discerns data to formulate a diagnostic impression/plan	□ Manages and prioritizes critical actions in a critically ill patient Reassesses after implementing a stabilizing intervention
2	**Performance of focused history and physical (PC2)**	□ Did not achieve Level 1	□ Performs a reliable, comprehensive history and physical exam	□ Performs and communicates a focused history and physical exam based on chief complaint and urgent issues	□ Prioritizes essential components of history and physical exam given dynamic circumstances
3	**Diagnostic studies (PC3)**	□ Did not achieve Level 1	□ Determines the necessity of diagnostic studies	□ Orders appropriate diagnostic studies. Performs appropriate bedside diagnostic studies/procedures	□ Prioritizes essential testing Interprets results of diagnostic studies Reviews risks, benefits, contraindications, and alternatives to a diagnostic study or procedure
4	**Diagnosis (PC4)**	□ Did not achieve Level 1	□ Considers a list of potential diagnoses	□ Considers an appropriate list of potential diagnosis May or may not make correct diagnosis	□ Makes the appropriate diagnosis Considers other potential diagnoses, avoiding premature closure
5	**Pharmacotherapy (PC5)**	□ Did not achieve Level 1	□ Asks patient for drug allergies	□ Selects an medication for therapeutic intervention, consider potential adverse effects	□ Selects the most appropriate medication and understands mechanism of action, effect, and potential side effects Considers and recognizes drug-drug interactions
6	**Observation and reassessment (PC6)**	□ Did not achieve Level 1	□ Reevaluates patient at least one time during case	□ Reevaluates patient after most therapeutic interventions	□ Consistently evaluates the effectiveness of therapies at appropriate intervals
7	**Disposition (PC7)**	□ Did not achieve Level 1	□ Appropriately selects whether to admit or discharge the patient	□ Appropriately selects whether to admit or discharge Involves the expertise of some of the appropriate specialists	□ Educates the patient appropriately about their disposition Assigns patient to an appropriate level of care (ICU/Tele/Floor) Involves expertise of all appropriate specialists
9	**General Approach to Procedures (PC9)**	□ Did not achieve Level 1	□ Identifies pertinent anatomy and physiology for a procedure Uses appropriate Universal Precautions	□ Obtains informed consent Knows indications, contraindications, anatomic landmarks, equipment, anesthetic and procedural technique, and potential complications for common ED procedures	□ Determines a back-up strategy if initial attempts are unsuccessful Correctly interprets results of diagnostic procedure
20	**Professional Values (PROF1)**	□ Did not achieve Level 1	□ Demonstrates caring, honest behavior	□ Exhibits compassion, respect, sensitivity and responsiveness	□ Develops alternative care plans when patients’ personal beliefs and decisions preclude standard care
22	**Patient centered communication (ICS1)**	□ Did not achieve level 1	□ Establishes rapport and demonstrates empathy to patient (and family) Listens effectively	□ Elicits patient’s reason for seeking health care	□ Manages patient expectations in a manner that minimizes potential for stress, conflict, and misunderstanding. Effectively communicates with vulnerable populations, (at risk patients and families)
23	**Team management (ICS2)**	□ Did not achieve level 1	□ Recognizes other members of the patient care team during case (nurse, techs)	□ Communicates pertinent information to other healthcare colleagues	□ Communicates a clear, succinct, and appropriate handoff with specialists and other colleagues Communicates effectively with ancillary staff

## INSTRUCTOR MATERIALS

**Case Title:** Eclampsia

**Case Description & Diagnosis (short synopsis):** A 24-year-old female with no known past medical history presents to the emergency department after having a witnessed seizure at home. Paramedics state the patient complained to her family about facial twitching, and then her whole body started shaking (but stops on arrival). Paramedics were not able to establish any IV lines or get the vital signs. She is now confused. The learner should first perform the primary survey to assess the stability of the patient. Then, the learner should obtain finger-stick blood glucose and perform a physical exam that reveals the patient is pregnant. The learner should recognize eclampsia and begin treatment with anti-hypertensive medications and magnesium sulfate. Simultaneously, learners should be able to consult the obstetrics and gynecology team for emergent evaluation and intervention of the patient, who will not be available temporarily. Monitoring and reversing magnesium toxicity are the final steps prior to disposition.

### Equipment or Props Needed

IV Supplies

- Angiocatheters
- IV tubing
- Saline

Airway Management

- Non-rebreather mask
- Nasal cannula
- Laryngoscope
- Bag-valve-mask
- Endotracheal tube and stylet
- Orogastric/nasogastric tube

Medications

- Etomidate
- Succinylcholine
- Epinephrine
- Atropine
- Labetalol
- Nicardipine
- Magnesium sulfate
- Hydralazine

Sim Exam

- Plain clothed SimMom mannequin or other gravid simulator (presenting via EMS)

Miscellaneous

- Gloves
- Alcohol/chloraprep
- Cardiac monitor

### Confederates needed

The simulation operator can be a pharmacist or consultant available via phone. The faculty member in the room can be utilized as a family if desired. With multidisciplinary teams, nursing can help with IV access and patient monitoring. Otherwise, nursing tasks can be delegated to a member of the resident team.

### Stimulus Inventory

#1 Complete blood count (CBC)
#2 Complete metabolic panel (CMP)
#3 Finger-stick blood glucose
#4 Venous blood gas
#5 Urinalysis/Urine drug screen/Urine Pregnancy
#6 Coagulation panel
#7 Fetal heart tracing
#8 Chest X-Ray
#9 Magnesium
#10 Brain CT
#11 Uric Acid/LDH/Haptoglobin
#12 Lactic Acid
#13: Repeat Magnesium: Pending
#14: Repeat Lactic Acid
#15: Arrival EKG
#16: Post-Magnesium EKG
#17: Phosphorus

**Background and brief information:** EMS bring a 24-year-old female to a tertiary medical center confused after what appeared to be a seizure.

**Initial presentation:** The patient arrives by EMS without family. She is an appropriately dressed female who has altered mental status.

**How the scene unfolds:** On arrival, learners should assess the patient’s airway, breathing, and circulation while obtaining any additional information from EMS before they leave. They should attempt to perform a history and focused physical exam on the patient who will be unable to provide any additional information because of altered mental status. They should recognize the patient is gravid, and obtain IV access while performing a finger-stick blood glucose which will be normal. They should notify obstetrics and gynecologists who will be unavailable for a short period and recommend the learner provide treatment. Learners should perform fetal heart monitoring while giving anti-hypertensive medications and magnesium sulfate. As the patient is monitored in the ED, the patient exhibits symptoms of magnesium toxicity and will require reversal with calcium. The case ends with disposition to the OR with OB.

### Critical actions

1. Place IV, obtain vitals and a finger-stick glucose
2. Perform primary survey (airway, breathing, circulation)
3. Obtain a basic history of symptoms
4. Perform physical exam and recognize pregnancy
5. Verbalize “eclampsia” and call obstetrics consult early
6. Emergent blood pressure management (labetalol or hydralazine)
7. Treat seizure with magnesium
8. Recognize magnesium toxicity
9. Provide antidote for magnesium toxicity
10. Clearly communicate with consultants and nurses with the management goals and disposition

**Case Title:** Eclampsia

**Chief Complaint:** Altered Mental Status

**Vitals:** Heart Rate (HR) 118 Blood Pressure (BP) 180/115 Respiratory Rate (RR) 24 Temperature (T) 99.0°F Oxygen Saturation (O_2_Sat) 95% on room air

**General Appearance:** Young, well-developed female, altered

**Primary Survey:**

- **Airway:** Patent
- **Breathing:** Clear to auscultation bilaterally
- **Circulation:** 2+ pulses throughout

### History

- **History of present illness:**
  ○ **Paramedic history:** Fire rescue states they were called to the scene for a witnessed seizure at home. Per family, the patient had some facial twitching, and then the patient’s whole body was shaking, and she was unresponsive for about 2 minutes. She was incontinent of urine at the scene. She has no prior history of seizures or any other medical problems. No known trauma.
  ○ **Patient history:** Patient unable to provide history secondary to altered mental status initially only stating “my head hurts.” However, as the case progresses with correct actions the patient will become more awake and will complain of a mild headache, blurring of vision, right upper quadrant pain, and bilateral lower extremity swelling.
  ○ If asked: The patient is 31weeks pregnant by dates and has only had 1 prenatal appointment in the first trimester but nothing since then.
- **Past medical history:** No chronic medical problems. First pregnancy (G1P0)
- **Past surgical history:** None
- **Patient’s medications:** Prenatal vitamins
- **Allergies:** None
- **Social history:** Drinks socially (last drink 6 months ago), no smoking or illicit drug use
- **Family history:** No history of seizures, intracranial aneurysms, or cancer

### Secondary Survey/Physical Examination

- **General appearance:** Postictal appearing, confused, sleepy, smells of urine, moaning and groaning
- **HEENT:**
  ○ **Head:** Within normal limits
  ○ **Eyes:** Pupils equally round and reactive 4mm ->2mm, no papilledema
  ○ **Ears:** No hemotympanum
  ○ **Nose:** Within normal limits
  ○ **Throat:** Clear oropharynx
- **Neck:** No stridor, trachea midline
- **Heart:** Tachycardic, regular rhythm, equal pulses, no murmurs
- **Lungs:** Clear to auscultation bilaterally, no respiratory distress, no wheezes/rhonchi/rales
- **Abdominal/GI:** Soft, mildly tender in the right upper quadrant, no distention, bowel sounds present, gravid uterus with fundus above umbilicus approximately 30 weeks, visible striae, no organomegaly
- **Genitourinary:** Within normal limits, no vaginal bleeding
- **Rectal:** Within normal limits
- **Extremities:** 1+ pitting edema in bilateral lower extremities, no muscle tenderness, full range of motion
- **Back:** Within normal limits
- **Neuro:** Glasgow Coma Scale (GCS)13, opens eyes to command, confused, follows commands). Oriented to self, pupils equally round and reactive, no facial droop, 3+ deep tendon reflexes, moving all extremities, able to squeeze hands equally, unable to further participate in the exam
- **Skin:** Normal color and turgor, no diaphoresis, no rash
- **Lymph:** Within normal limits
- **Psych:** Within normal limits

### Results

*Complete blood count (CBC)*

[Table t2-jetem-6-3-s33]

*Complete metabolic panel (CMP)* [Table t3-jetem-6-3-s33]

[Table t4-jetem-6-3-s33]

*Venous Blood Gas (VBG)* [Table t5-jetem-6-3-s33]

*Urinalysis (UA)* [Table t6-jetem-6-3-s33]

*Urine drug screen* [Table t7-jetem-6-3-s33]

[Table t8-jetem-6-3-s33]

*Coagulation Panel* [Table t9-jetem-6-3-s33]

*Fetal Heart Tracing*

Baseline fetal heart rate 130bpm with decrease in fetal heart rate from baseline lasting 30 seconds. Each decrease in fetal heart rate occurs after each contraction concerning for late decelerations[Fig f2-jetem-6-3-s33]

*Chest Radiograph (CXR)*

No acute disease in the chest[Table t10-jetem-6-3-s33]

*Brain Computed Tomography*

No acute intracranial abnormalities[Table t11-jetem-6-3-s33]

*Arrival Electrocardiogram (EKG)*

File NSR (CardioNetworks ECGpedia) is licensed under creative commons attribution-share alike 3.0 imported (CC BY-SA 3.0)[Fig f3-jetem-6-3-s33]

*Post-Magnesium Electrocardiogram (EKG)* [Fig f4-jetem-6-3-s33]

[Table t12-jetem-6-3-s33]

## OPERATOR MATERIALS

[Table t13-jetem-6-3-s33]

### Diagnosis

Eclampsia

### Disposition

Admission to the ICU

## DEBRIEFING AND EVALUATION PEARLS

### Eclampsia

#### Eclampsia: Definition, Pathophysiology

Eclampsia is one of the obstetrics emergency clinicians should be familiar with because it carries one of the highest mortality and morbidity rates for both the mother and the baby.^10^ It causes a mortality rate of 14% worldwide.^4^ According to the American Board of Emergency Medicine Model of Practice, pre-eclampsia and eclampsia are included as one of the critical pathologies emergency physicians should be able to manage.^11^ Thus, it is essential for Emergency Medicine residents to be familiar with the recognition and the management of eclampsia.

Eclampsia is defined as “the occurrence of one or more generalized tonic-clonic convulsions unrelated to other medical conditions in women with hypertensive disorder of pregnancy.” 1 Although it is not precisely known, there are a few hypotheses why this disorder occurs. One of the theories involves the change in autoregulation in the central nervous system and the brain-blood barrier. Another theory involves the overregulation of the central nervous system and its result of vasoconstriction.^12^

#### Eclampsia: Presentation, “It is a spectrum.”

Emergency providers have difficulty recognizing this obstetrical emergency because of its atypical presentation and its wide spectrum of preeclampsia to eclampsia. Preeclampsia can also be divided further into a mild form and a severe form based on severe features and abnormal lab parameters.

Chronic hypertension is the elevated blood pressure of at least 140/90 on 2 separate occasions at least 4 hours apart diagnosed before 20 weeks of gestation, while gestational hypertension is defined as high blood pressure after 20 weeks of gestations without proteinuria and without any features or preeclampsia. This is also important because about half of patients with gestational hypertension may develop preeclampsia or eclampsia.^13^

The mild form of preeclampsia is defined as gestational hypertension plus either of the following:

- Evidence of proteinuria defined as>300mg/24hour urine specimen (including 2 dipstick readings>2+ taken 6 hours apart), or protein to creatinine ratio >0.3.^13^

However, preeclampsia criteria is different in patients with chronic hypertension. About 20 to 50% of patients with chronic hypertension may develop preeclampsia or eclampsia. 13 It is defined as chronic hypertension, where the systolic blood pressure must have increased by 30mmHg or diastolic blood pressure increased by 15mmHg with mild proteinuria (as defined above).^1^ ^13^

The severe form differs from the mild form with a higher blood pressure parameter and severe features. First, the blood pressure should be greater than or equal to the systolic blood pressure of 160 mmHg or the diastolic blood pressure of 110mmHg or higher on two separate occasions at least 4 hours apart on bed rest.^13^ Some severe feature findings include a) pulmonary edema or hypoxia, b) renal insufficiency, serum creatinine >1.1 mg/dL or doubling creatinine in the absence of kidney disease, c) new-onset headache, d) presence of visual disturbances, e) persistent epigastric or right upper quadrant pain, f) impaired liver function tests, g) generalized weakness, h) bleeding at intravenous insertions, i) thrombocytopenia <100,000 and j) altered mental state.^14^

Eclampsia differs from preeclampsia because it is the convulsive presentation of hypertensive obstetrical emergency.^13^ Seizures can manifest with tonic-clonic, focal or multifocal seizures without a history of seizures.

Despite the differences in definition, preeclampsia and eclampsia can present any time between 20 weeks of gestation to 4 weeks postpartum. These hypertensive emergencies should be recognized and managed promptly since 1 in 400 women with preeclampsia with mild features and 1 in 50 women with preeclampsia with severe features develop eclampsia, which remains a significant cause of maternal death worldwide.^13^

A prompt recognition and management of this obstetrical emergency is paramount to decrease the morbidity and mortality associated with the condition.

#### Pre-Eclampsia & Eclampsia: Management Part 1: Seizure Prophylaxis and Seizure treatment

In an emergency, physicians should resuscitate with airway, breathing, and circulation before further management or investigation of the patient.

The management of seizure can be divided into two-folds: prophylaxis and treatment.

The seizure prophylaxis is indicated in patients with preeclampsia with severe features. Since these patients’ likelihood of developing into eclampsia is high, the American College of Obstetrics and Gynecology recommends magnesium load.^13^ It is important to be familiar with the dosing of magnesium. Magnesium is loaded with 4-to-6 grams 10% magnesium sulfate in 100ml solution via IV over 15 minutes. Then, a continuous infusion of 1–2 grams/hour should follow. Studies have not demonstrated any mortality or morbidity benefit in patients with preeclampsia with mild features.^1^

Once the patient is actively seizing in the scenario, it is crucial to clear the secretions and prevent asphyxiation and aspiration. After that, seizure should be aborted as soon as possible. Learners should realize that the first line of seizure treatment in eclampsia is not anticonvulsants but rather magnesium. The dosing is the same as the seizure prevention. If seizures recur in real-life settings, physicians can give an additional dose of 2g bolus of magnesium sulfate.^1^ However, physicians should start to think of alternative diagnoses and administer anticonvulsants, lorazepam, or phenytoin, if seizures are refractory to eclampsia’s corrective measures.

The dosing of magnesium is significantly higher than what is typically used to treat other pathology. This may lead to some confusion for nurses where they may not feel comfortable administering the medication. Thus, it is essential to communicate the indication, the reason, and side effects to monitor for the medication. Symptoms of hypermagnesemia can be rapid and start to manifest around the level of 3.5. Symptoms observed include nausea and somnolence (level 3–4), the loss of deep tendon reflex and muscle weakness (level 4–8), respiratory failure (level 8–12), hypotension, bradycardia and cardiac collapse (level 12–15). EKG changes (prolonged PR, increased duration of QRS complex, prolonged QTc, increased amplitude of T waves and delayed conduction) vary and do not correlate with the level of magnesium. If patients have a normal patellar reflex, respiratory rate >12, and urine output >100cc, the magnesium maintenance therapy can be continued.^15^ If any side effects occur, physicians should stop the medication and give the antidote, calcium gluconate 10% 1.5–3g, via IV. Also, since the medication is cleared via kidneys, the medication dose should be adjusted in renal failure patients—furthermore, some relative contraindications of the medication in myasthenia gravis, pulmonary edema, and renal edema.^16^

Besides the prophylaxis and chemical treatment, learners should also emergently consult the obstetrics team for definitive eclampsia management: delivery. However, learners need to understand that the patient should be resuscitated and free of seizure before transport to the delivery room.

#### Pre-Eclampsia & Eclampsia: Management Part 2: Calling Consultants

Obstetricians should be called emergently with patients who present with both preeclampsia and eclampsia. This is to evaluate the fetus for fetal heart rate and monitoring and assess the need for an emergent delivery because it is the most definitive treatment of preeclampsia and eclampsia. Especially to patients who present with preeclampsia with severe features after 34 weeks of gestation, there are delivery criteria that obstetricians will look for: non-reassuring fetal heart activity, uncontrollable blood pressure, oliguria, markedly elevated creatinine, pulmonary edema, hypoxia, development of HELLP (hemolysis, elevated liver enzymes, low platelets), right upper quadrant tenderness, or any pregnancy complications such as ruptured membranes, oligohydramnios, intrauterine growth restriction. Most of the time, emergency providers will not have any supporting documents, such as lab values, because it takes time for returns. However, informing obstetricians of a potential obstetrics emergency based on other collaterals such as vital signs or symptoms can alert them to evaluate the patient and the fetus emergently.^7^

When calling consultants, learners need to be concise and direct. There is the “5C’s” model, which stands for: contact, communicate, core question, collaborate, and close the loop. This model was extensively studied in medical students but had the most extensive evidence of making a high-quality consult.^17^ Besides, learners should include pertinent information specific to each specialty. For example, in this simulated case, learners should include gravida/para status, gestational week, presenting issue, ultrasound/lab results (if present), and the last oral intake, since there might be a need for the patient to go to the operating room.

#### Pre-Eclampsia & Eclampsia Management Part 3: Blood Pressure Management

Since the definition of preeclampsia and eclampsia entails hypertension, learners should not only recognize but also correctly measure blood pressure. The blood pressure should be taken in either a sitting or semi-reclining position on the right arm with the right size cuff. Once learners realize that it is true hypertension, then there should be a few safe and effective anti-hypertensive agents that should come to mind to utilize in pregnant patients. Several medications can be used to lower the blood pressure effectively: hydralazine, labetalol, nifedipine, nicardipine, nitroglycerine, or nitroprusside. Although effective, providers should be cautious of the side effects medications can manifest. Labetalol is cautioned against patients with low heart rates because it blocks the atrioventricular node of the heart.

Hydralazine should be cautioned to use in renal patients because it is cleared more slowly in patients with acute kidney injury. Also, it is longer acting, so providers should be careful in redosing if blood pressure does not effectively reduce right away. Some agents like nitroprusside or nitroglycerine can be used; however, they can induce cyanide toxicity, so additional caution must be addressed. Since there is no consensus on the first-line agent, any available medications can be used.^13^ However, it is probably best to choose an agent that is familiar to the nursing team. Also, learners should communicate with nurses the goal and the titration of anti-hypertensives, especially if selecting a drip form. The goal of medication for severe preeclampsia is to lower the systolic blood pressure to less than 160 mmHg and diastolic to less than 110 mmHg to prevent progression to severe complications.^13^

### Other debriefing points

Besides the case’s flow and medical management, debriefing facilitators should also focus on teamwork and communication to cover attitudes.

Communication

- Was there a clear communication between the standardized patient and the participant?
- Was there a clear consultation between the participant and the consultant?

Teamwork

- Was there close-loop communications among team members?
- Was there a shared mental model among team members?

**Wrap Up:** Emergency medicine textbooks on preeclampsia as well as the American College of Obstetricians and Gynecologists can provide additional material for interested learners.

## SIMULATION ASSESSMENT

*Eclampsia*

Learner: _________________________________________

***Assessment Timeline***

This timeline is to help observers assess their learners. It allows observer to make notes on when learners performed various tasks, which can help guide debriefing discussion.

[Table t14-jetem-6-3-s33]

### Critical Actions

□ Place IV, obtain vitals and a finger-stick glucose
□ Perform primary survey (airway, breathing, circulation)
□ Obtain a basic history of symptoms
□ Perform physical exam and recognize pregnancy
□ Verbalize “eclampsia” and call obstetrics consult early
□ Emergent blood pressure management (labetalol or hydralazine)
□ Treat seizure with magnesium
□ Recognize magnesium toxicity
□ Provide antidote for magnesium toxicity
□ Clearly communicate with consultants and nurses with the management goals and disposition

**Summative and formative comments:**

**Milestones assessment:** [Table t15-jetem-6-3-s33]
