# Supplementary material for: Eclampsia
Source: J Educ Teach Emerg Med. 2021 Jul 15;6(3):S33–61. doi: 10.21980/J8PS8R (PMC10332685; doi:10.21980/J8PS8R)
Supplement: Supplementary file 1 [file jetem-6-3-s33-supp.pptx]

## Slide 1
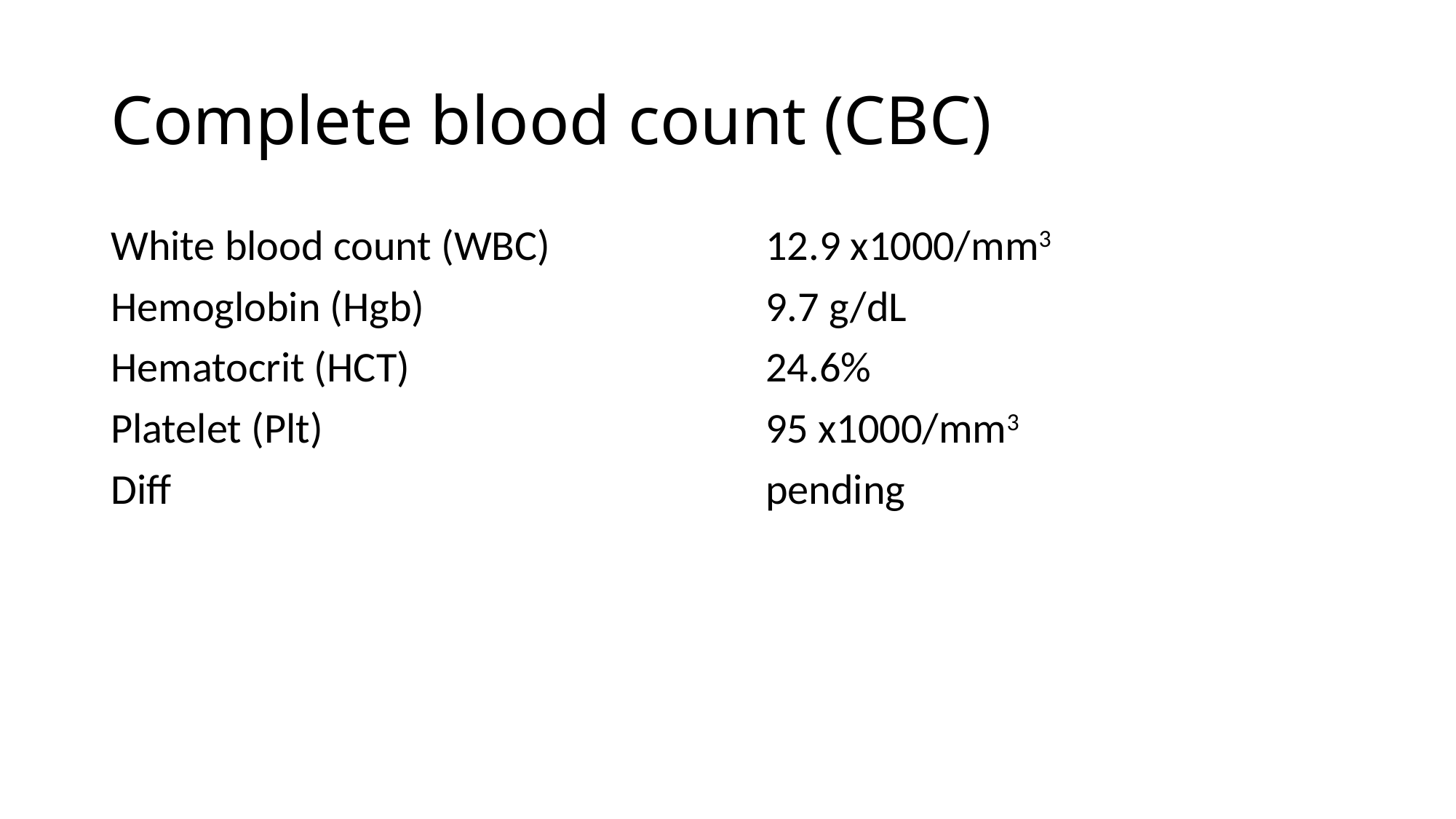

# Complete blood count (CBC)
White blood count (WBC) 		12.9 x1000/mm3
Hemoglobin (Hgb)				9.7 g/dL
Hematocrit (HCT)				24.6%
Platelet (Plt) 				95 x1000/mm3
Diff						pending

## Slide 2
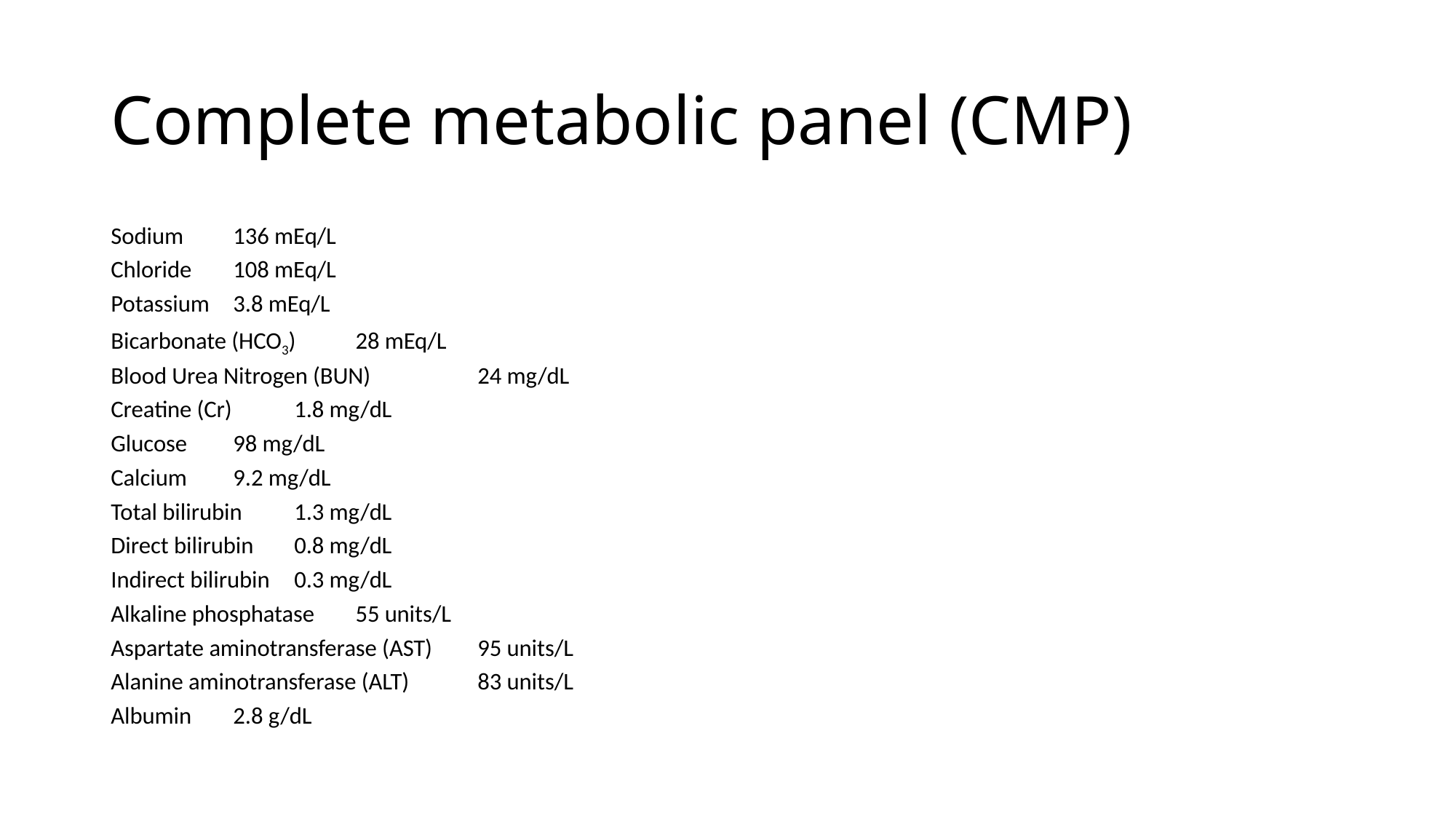

# Complete metabolic panel (CMP)
Sodium 					136 mEq/L
Chloride 					108 mEq/L
Potassium					3.8 mEq/L
Bicarbonate (HCO3)				28 mEq/L
Blood Urea Nitrogen (BUN)			24 mg/dL
Creatine (Cr)		 		1.8 mg/dL
Glucose 					98 mg/dL
Calcium					9.2 mg/dL
Total bilirubin				1.3 mg/dL
Direct bilirubin				0.8 mg/dL
Indirect bilirubin				0.3 mg/dL
Alkaline phosphatase				55 units/L
Aspartate aminotransferase (AST)			95 units/L
Alanine aminotransferase (ALT)			83 units/L
Albumin					2.8 g/dL

## Slide 3
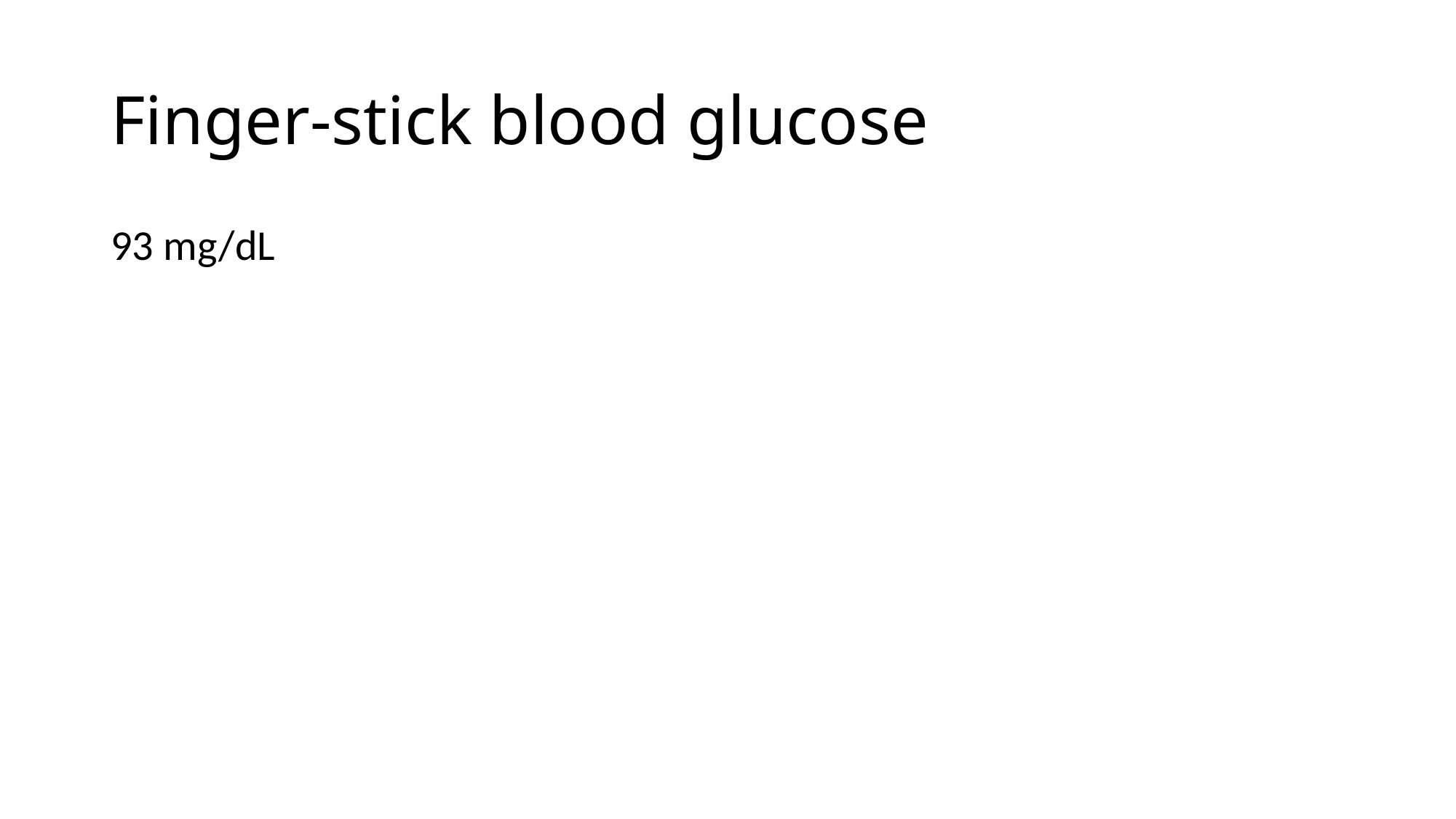

# Finger-stick blood glucose
93 mg/dL

## Slide 4
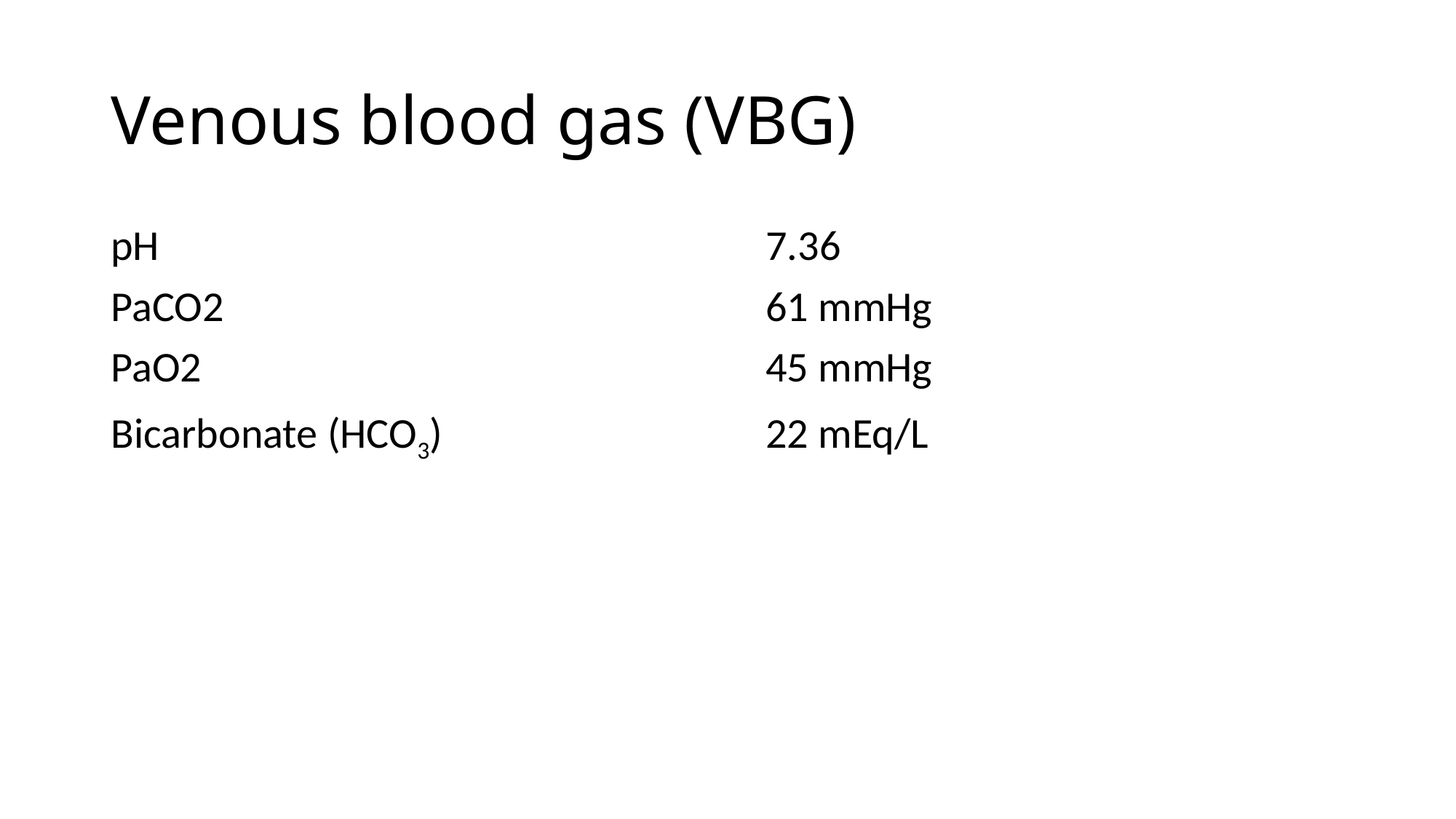

# Venous blood gas (VBG)
pH						7.36
PaCO2					61 mmHg
PaO2						45 mmHg
Bicarbonate (HCO3)			22 mEq/L

## Slide 5
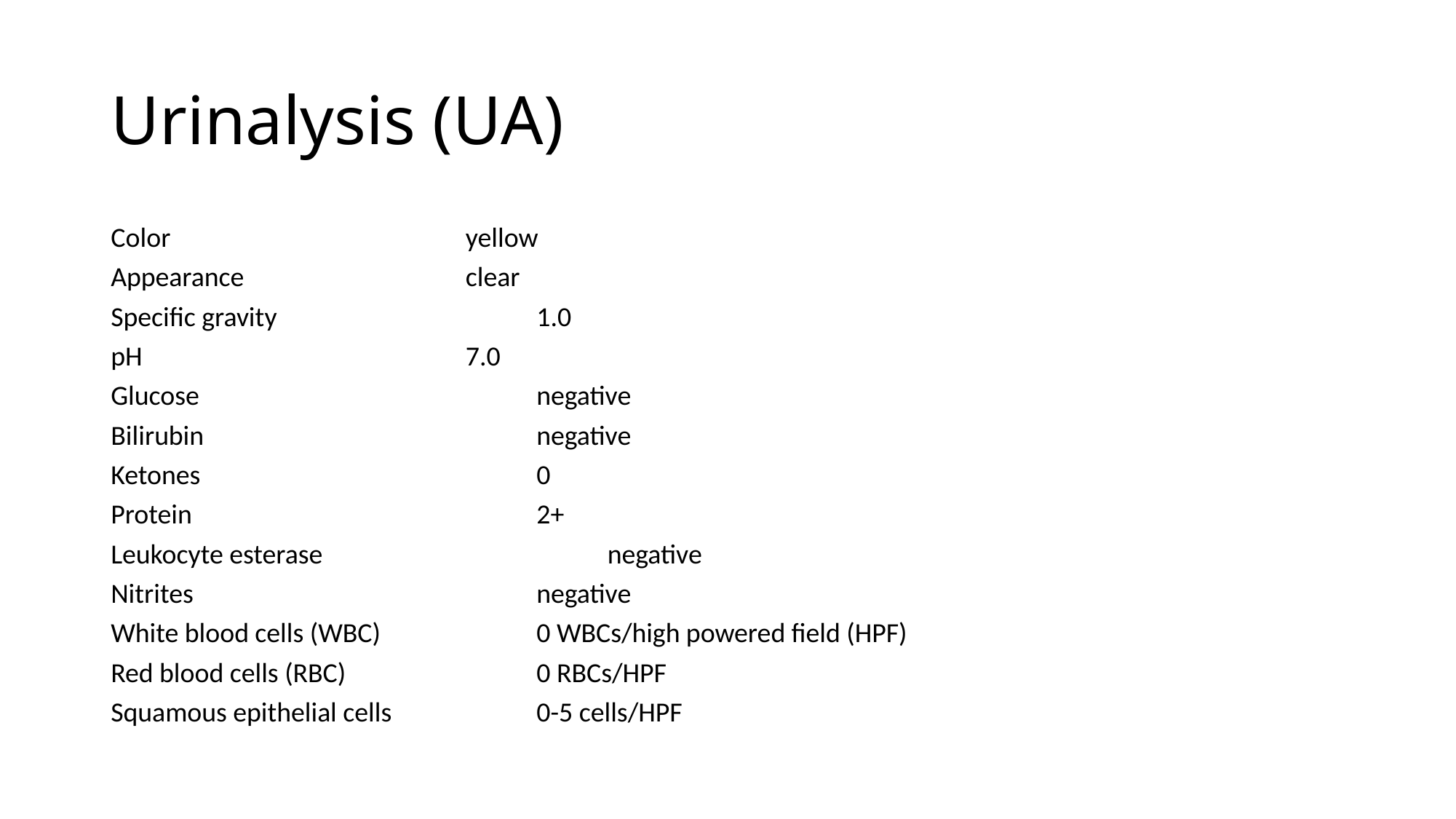

# Urinalysis (UA)
Color					yellow
Appearance				clear
Specific gravity				1.0
pH					7.0
Glucose					negative
Bilirubin					negative
Ketones					0
Protein					2+
Leukocyte esterase 				negative
Nitrites					negative
White blood cells (WBC)			0 WBCs/high powered field (HPF)
Red blood cells (RBC)			0 RBCs/HPF
Squamous epithelial cells			0-5 cells/HPF

## Slide 6
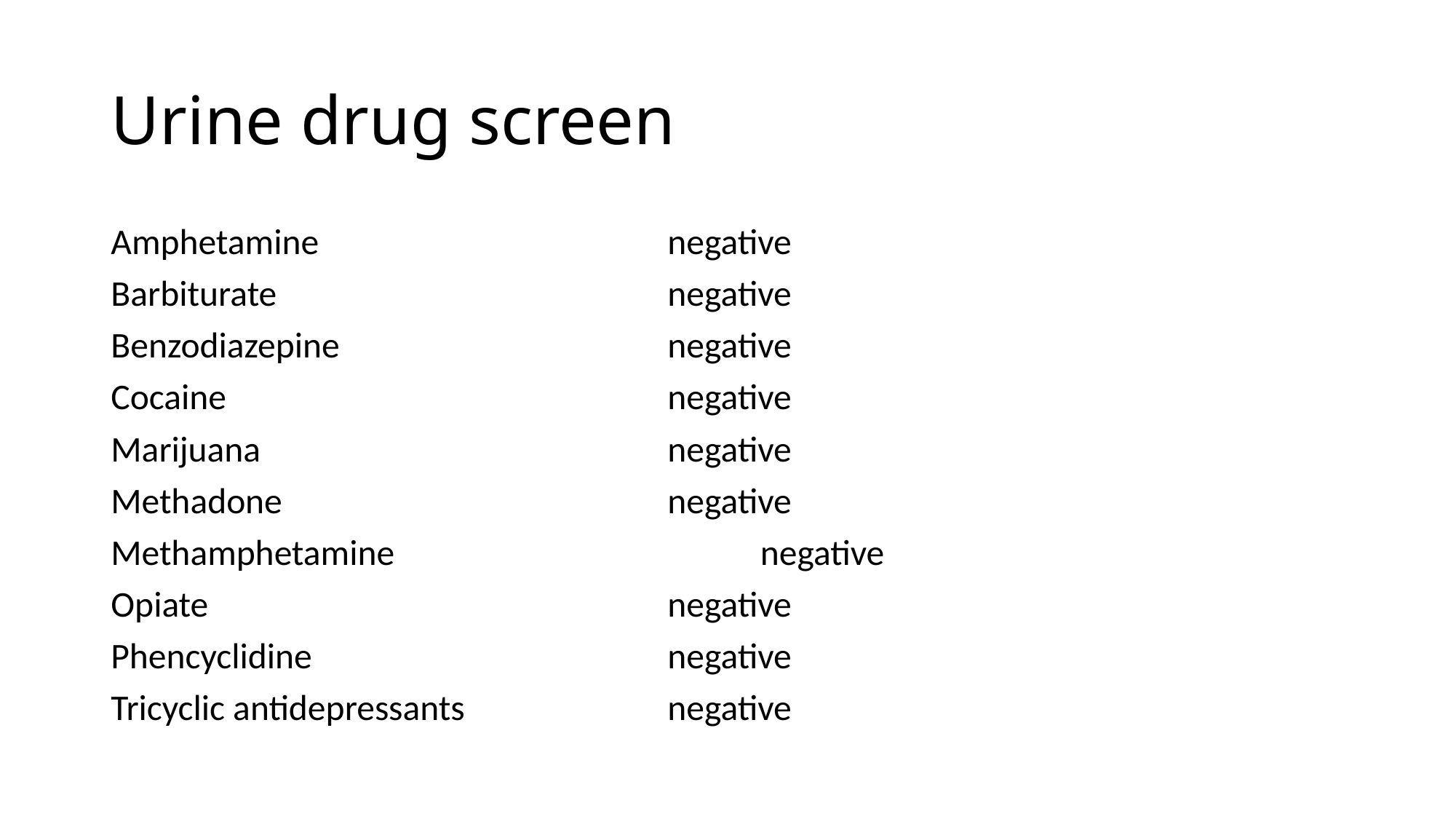

# Urine drug screen
Amphetamine 				negative
Barbiturate 					negative
Benzodiazepine 				negative
Cocaine 					negative
Marijuana 					negative
Methadone 					negative
Methamphetamine				negative
Opiate 					negative
Phencyclidine 				negative
Tricyclic antidepressants 			negative

## Slide 7
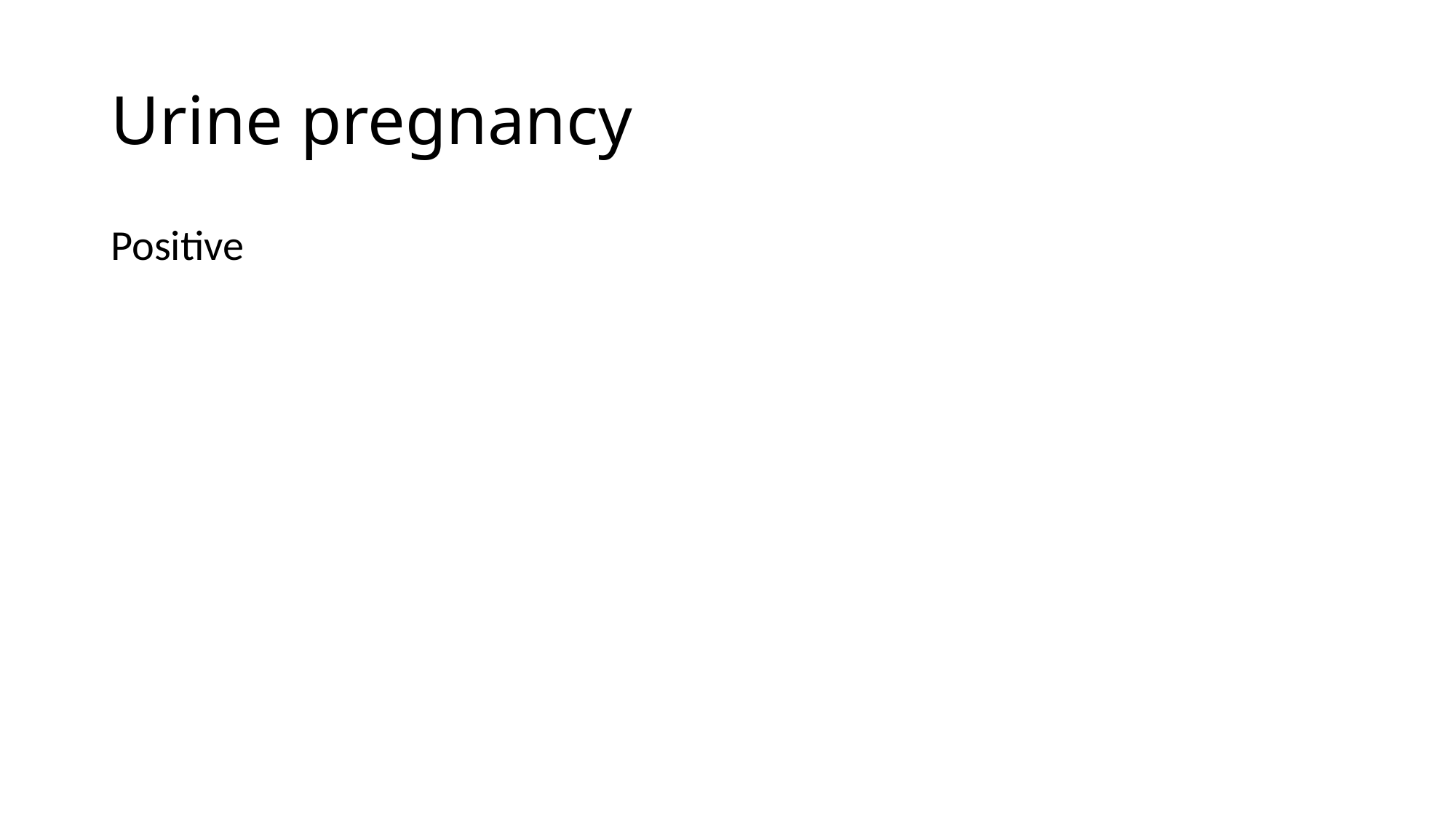

# Urine pregnancy
Positive

## Slide 8
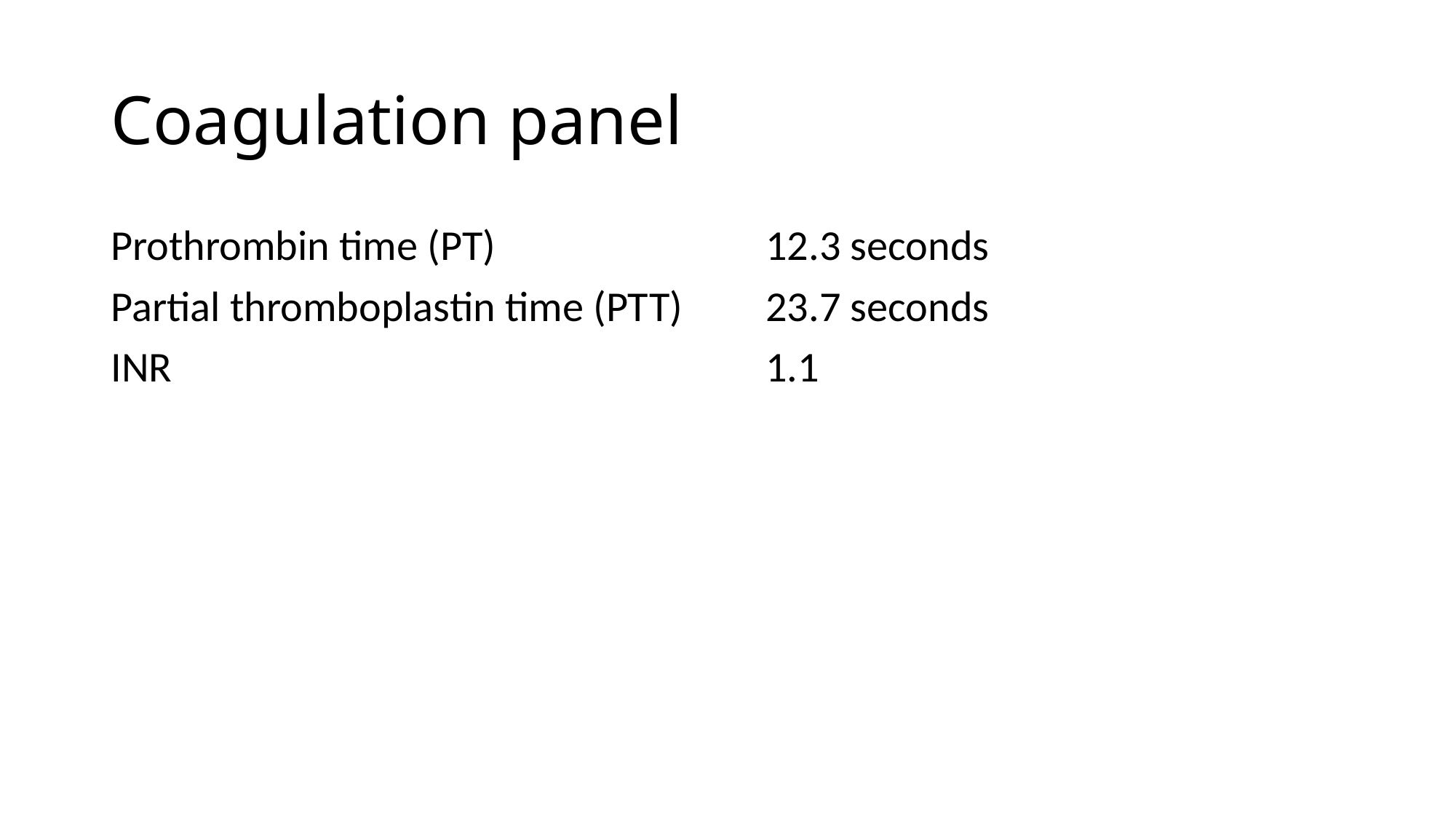

# Coagulation panel
Prothrombin time (PT) 			12.3 seconds
Partial thromboplastin time (PTT) 	23.7 seconds
INR 						1.1

## Slide 9
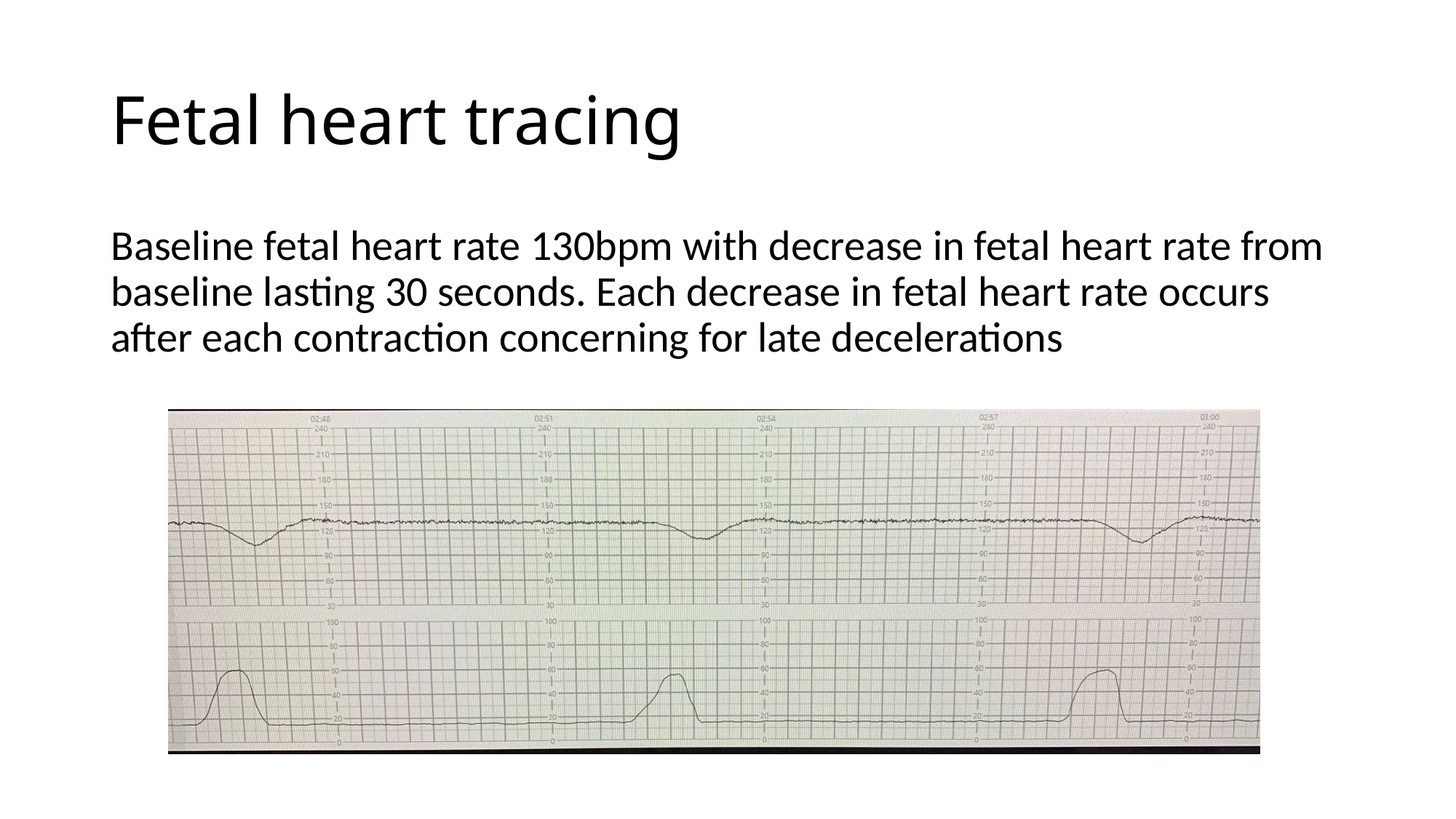

# Fetal heart tracing
Baseline fetal heart rate 130bpm with decrease in fetal heart rate from baseline lasting 30 seconds. Each decrease in fetal heart rate occurs after each contraction concerning for late decelerations

## Slide 10
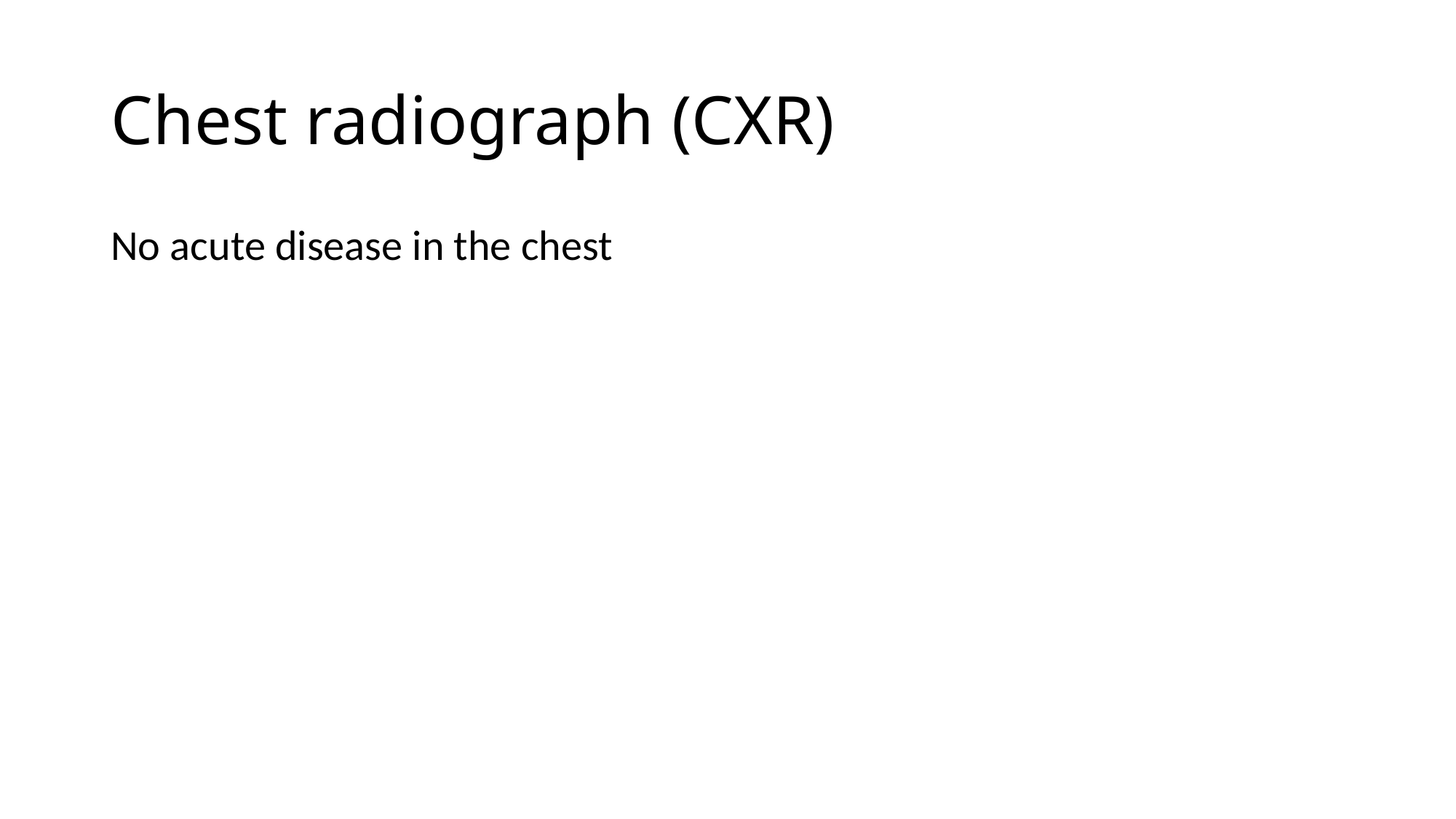

# Chest radiograph (CXR)
No acute disease in the chest

## Slide 11
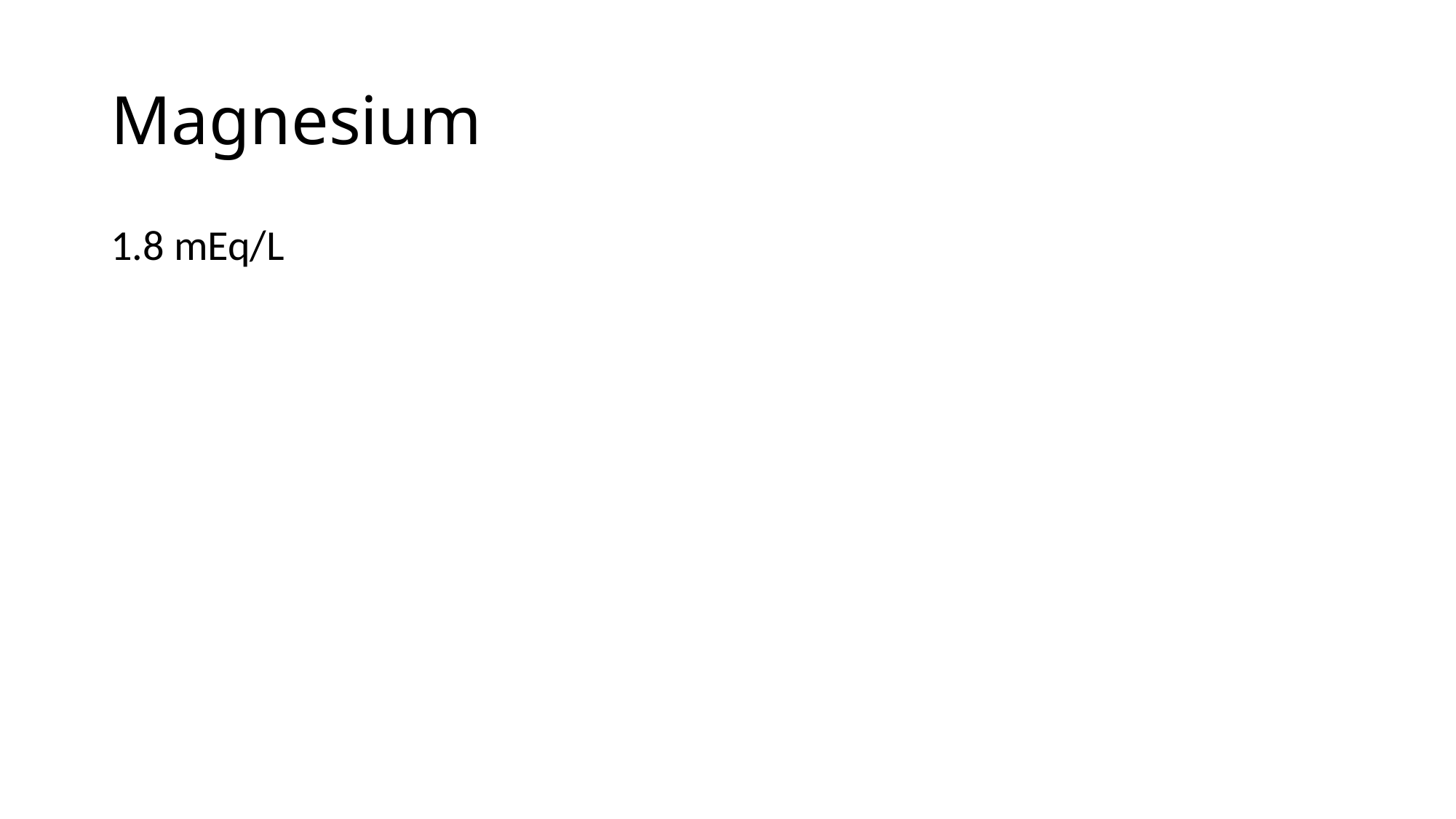

# Magnesium
1.8 mEq/L

## Slide 12
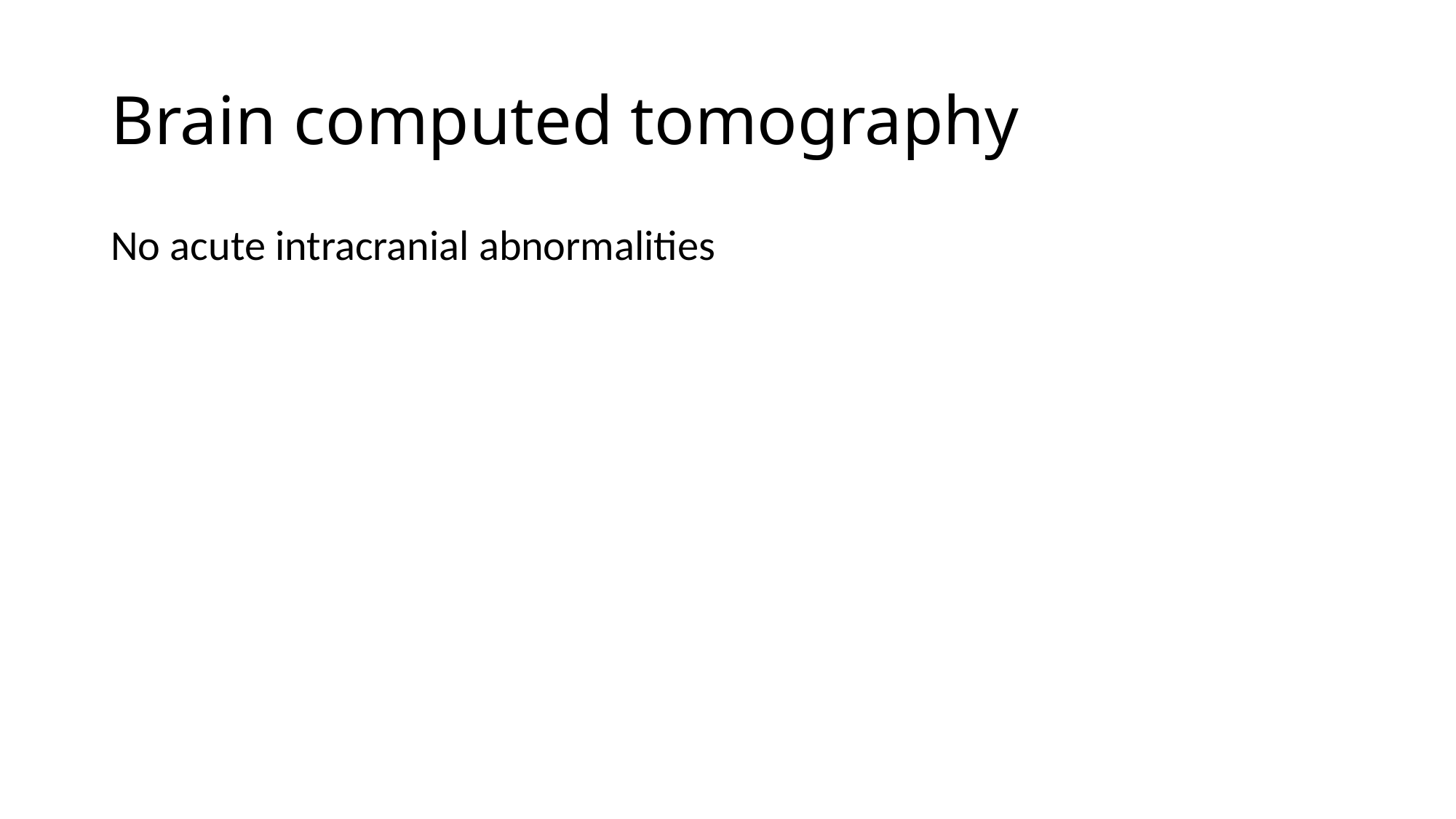

# Brain computed tomography
No acute intracranial abnormalities

## Slide 13
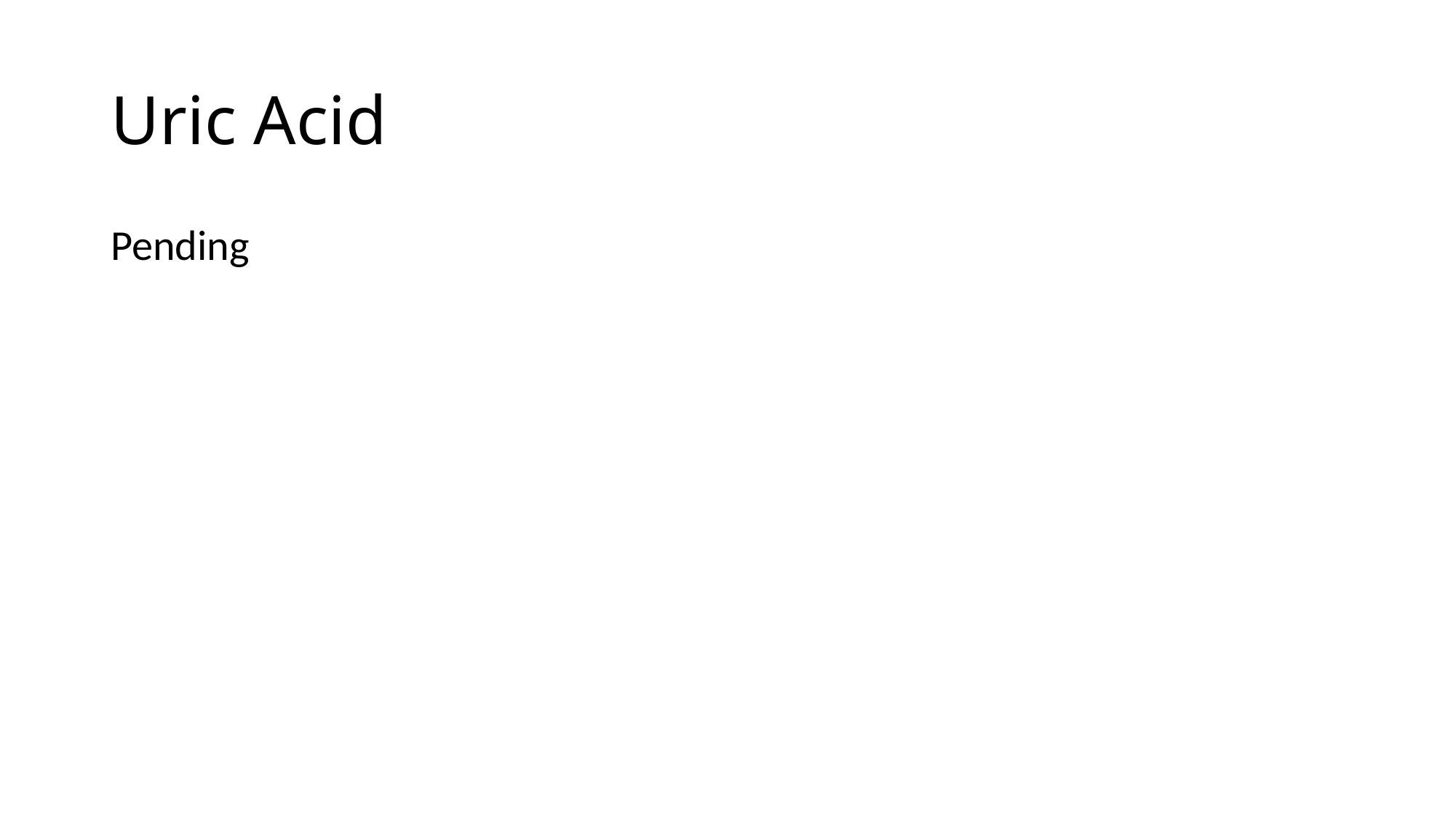

# Uric Acid
Pending

## Slide 14
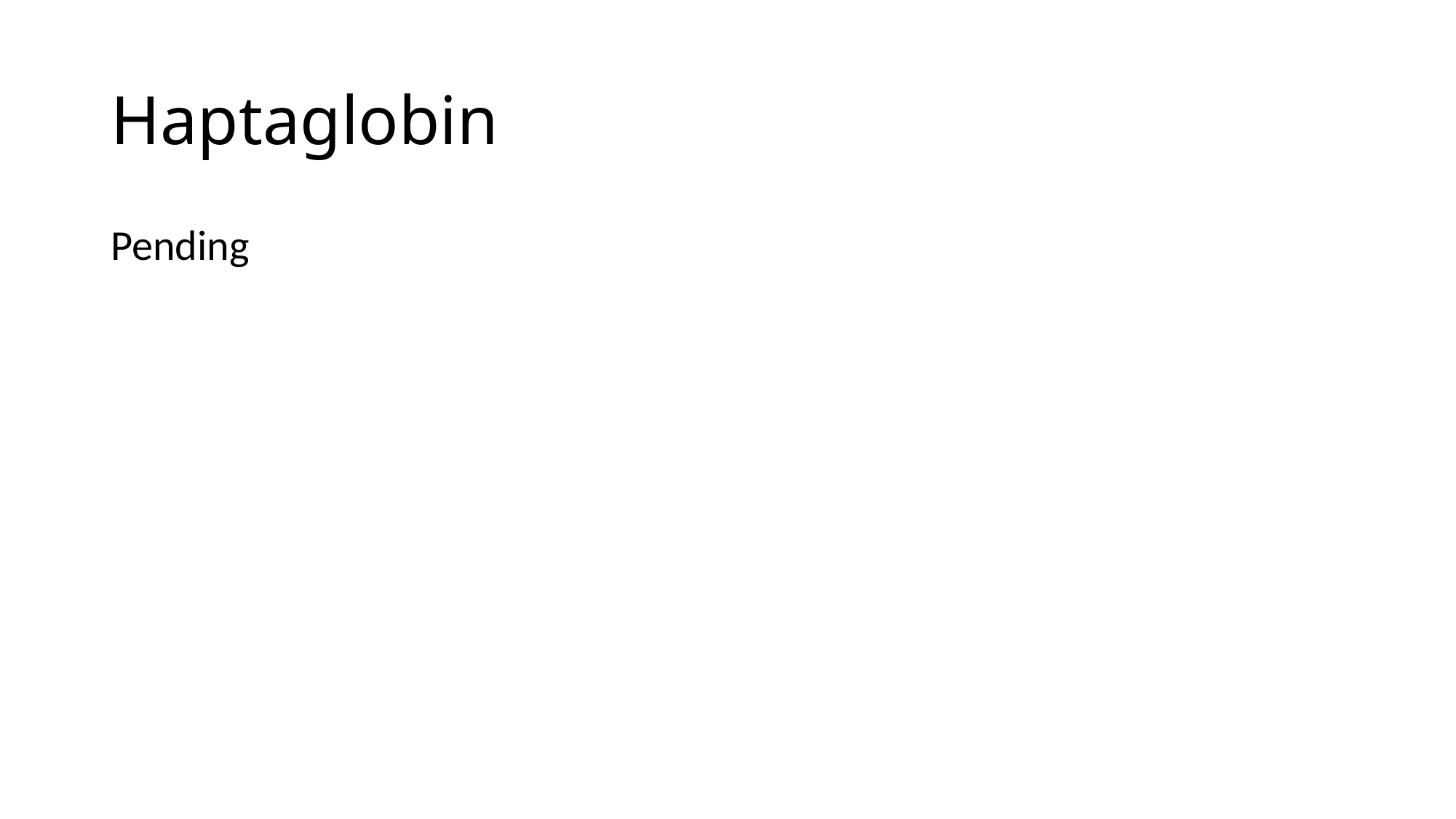

# Haptaglobin
Pending

## Slide 15
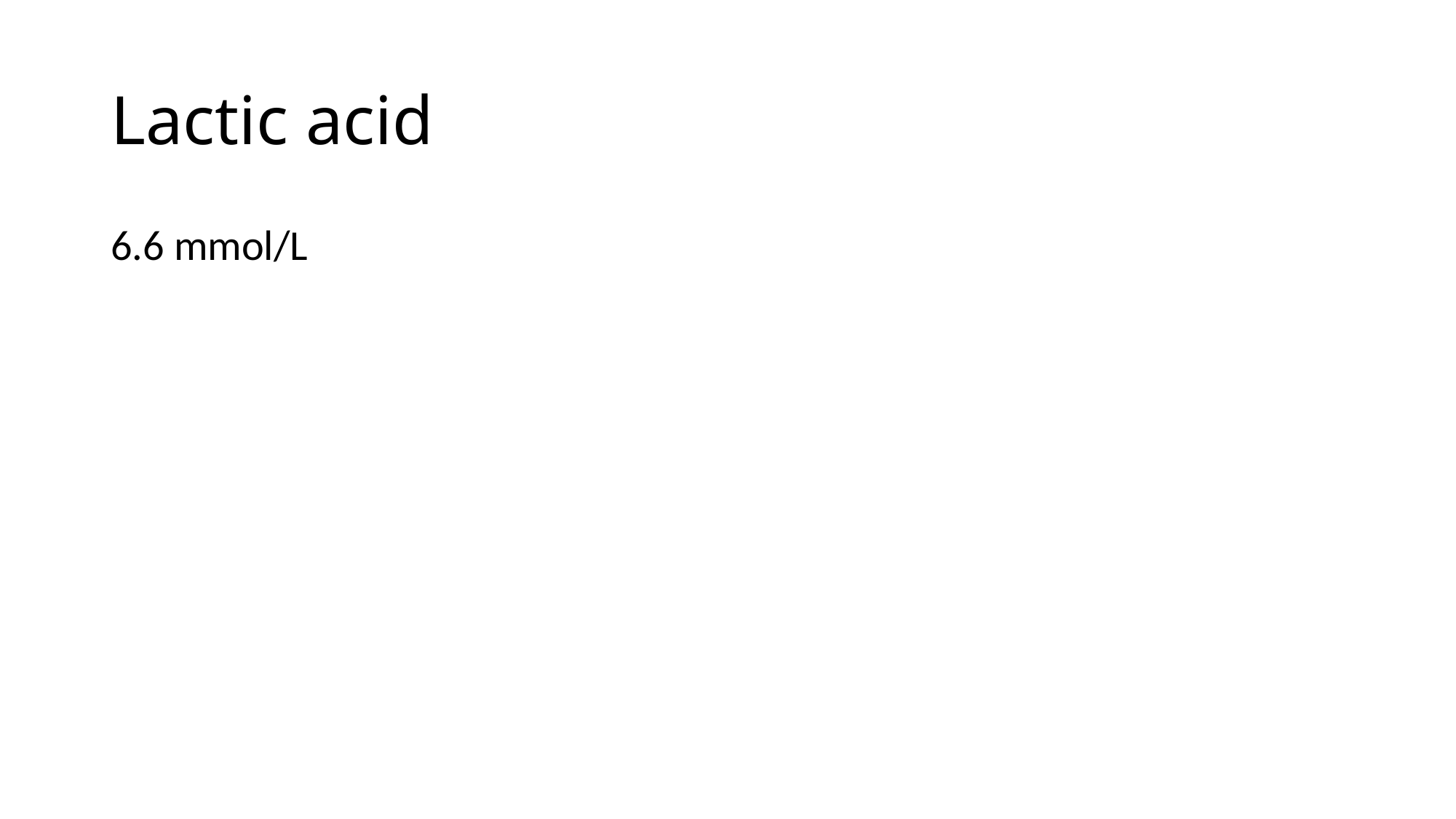

# Lactic acid
6.6 mmol/L

## Slide 16
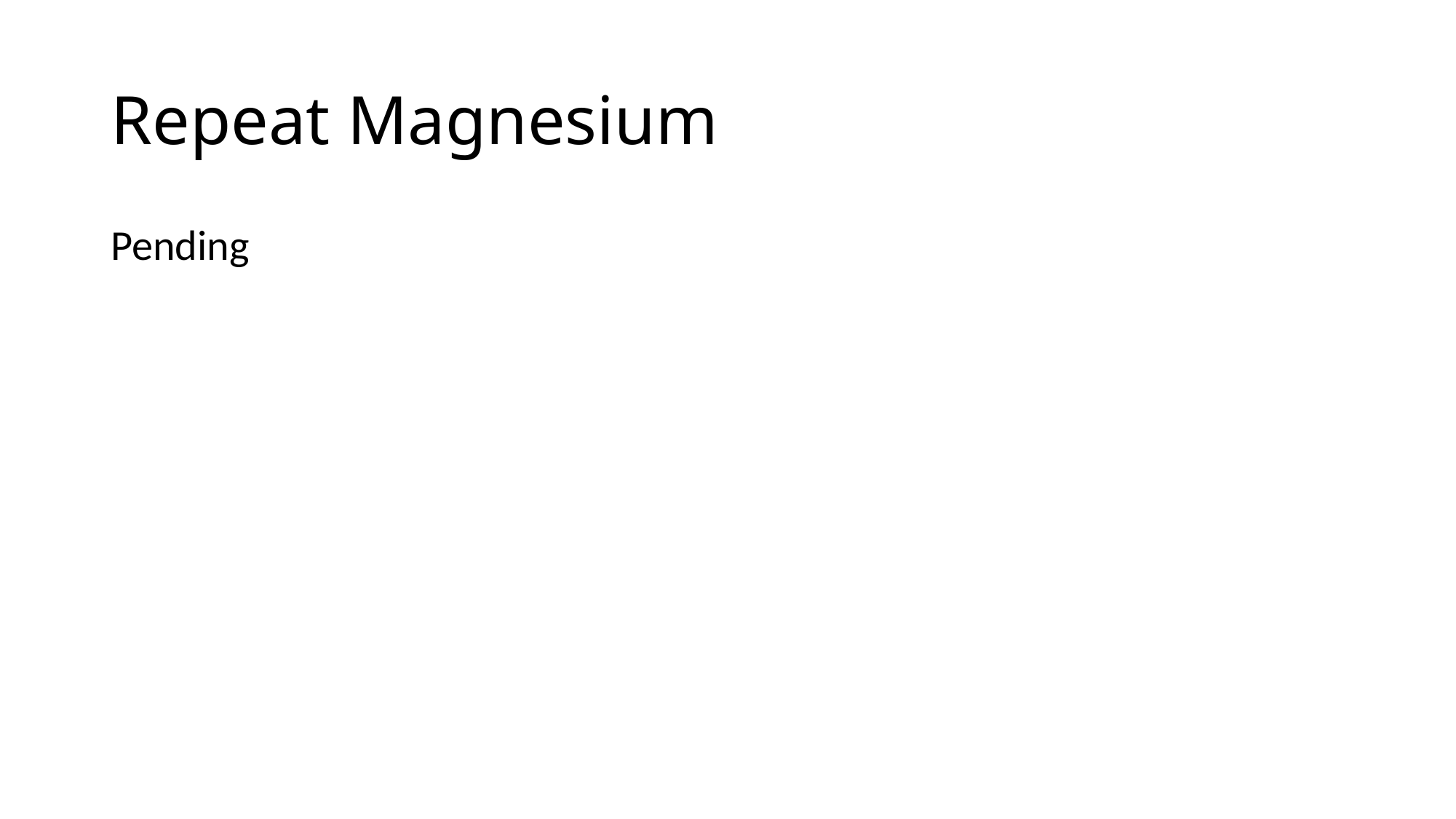

# Repeat Magnesium
Pending

## Slide 17
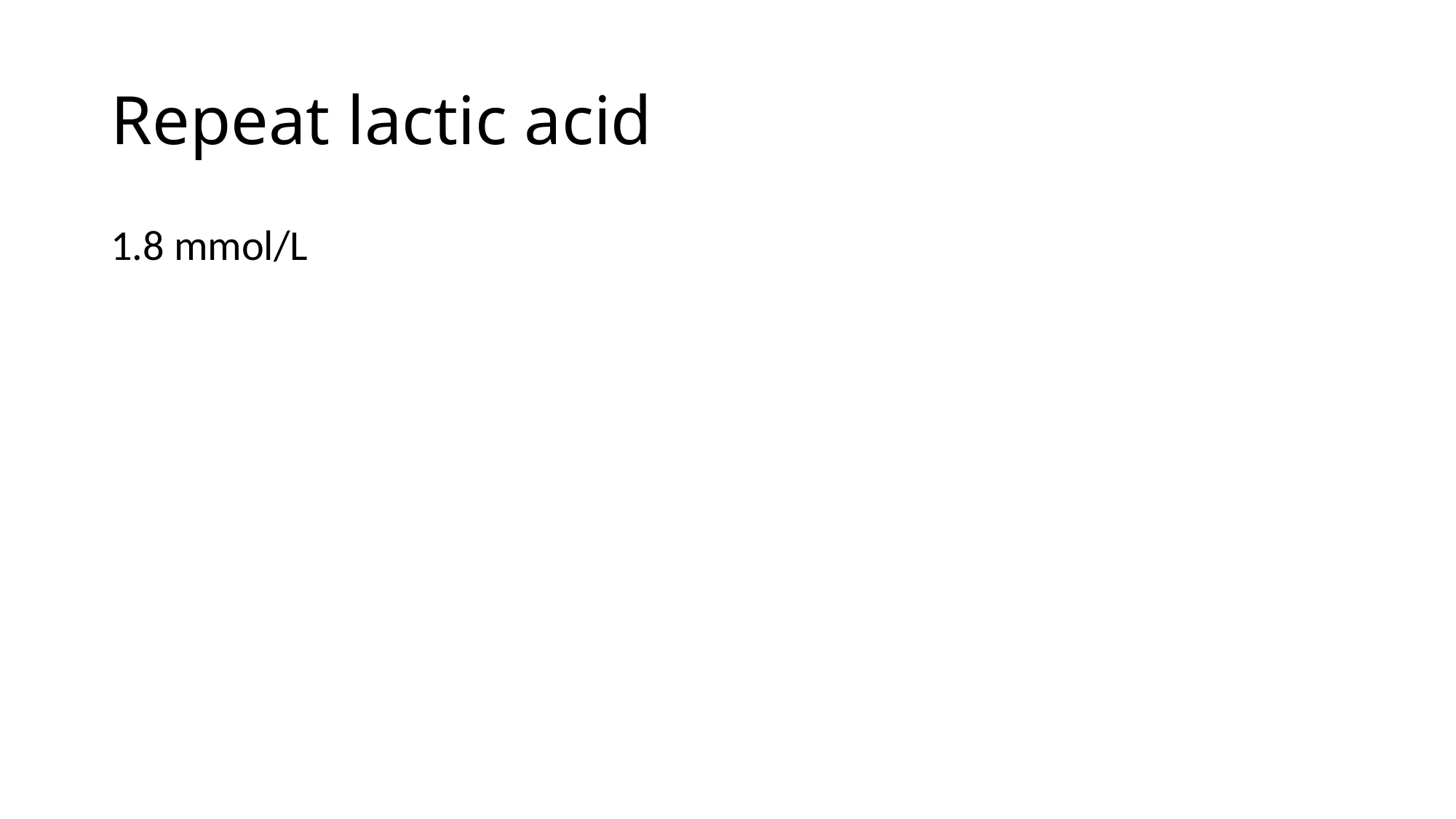

# Repeat lactic acid
1.8 mmol/L

## Slide 18
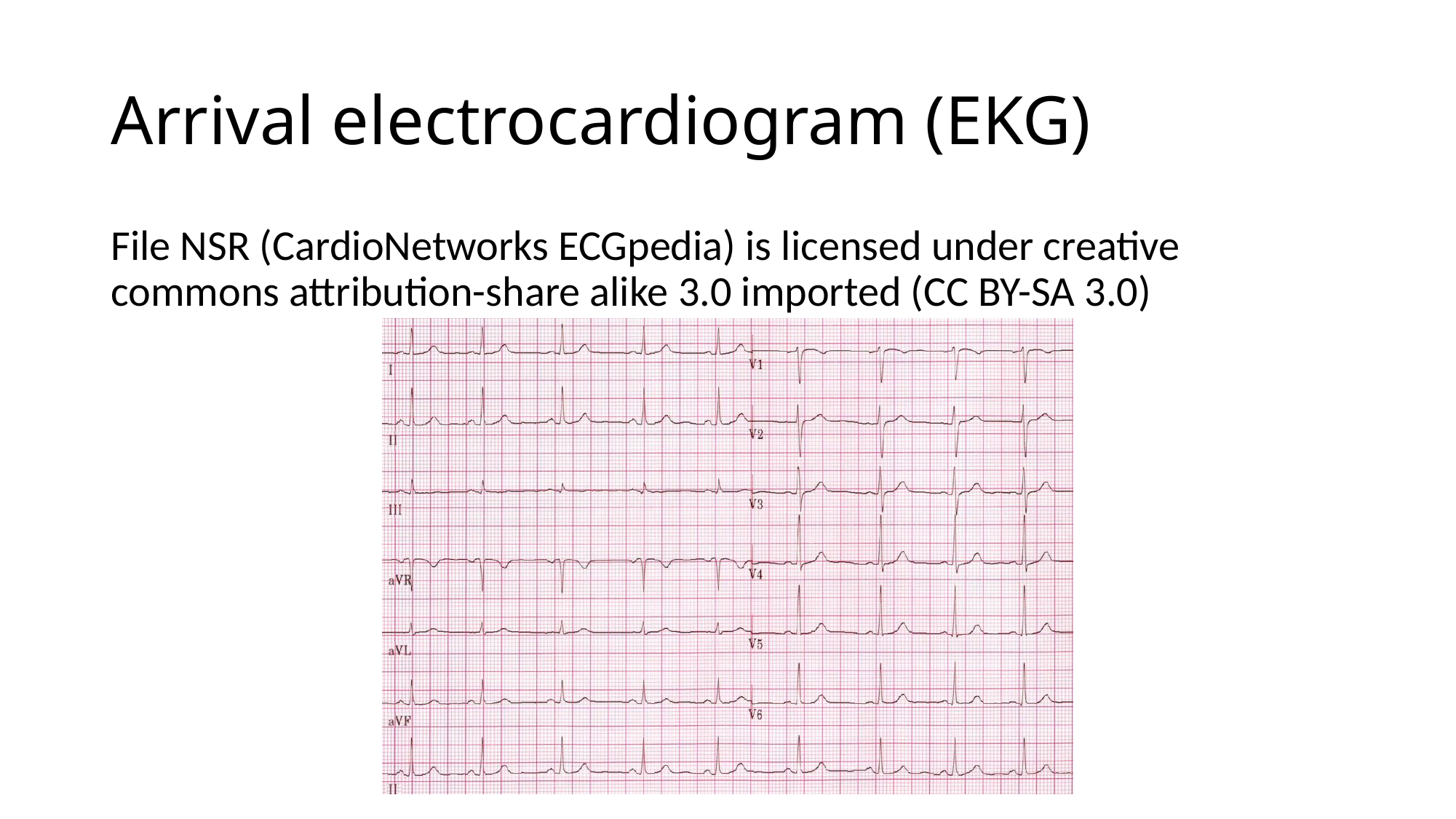

# Arrival electrocardiogram (EKG)
File NSR (CardioNetworks ECGpedia) is licensed under creative commons attribution-share alike 3.0 imported (CC BY-SA 3.0)

## Slide 19
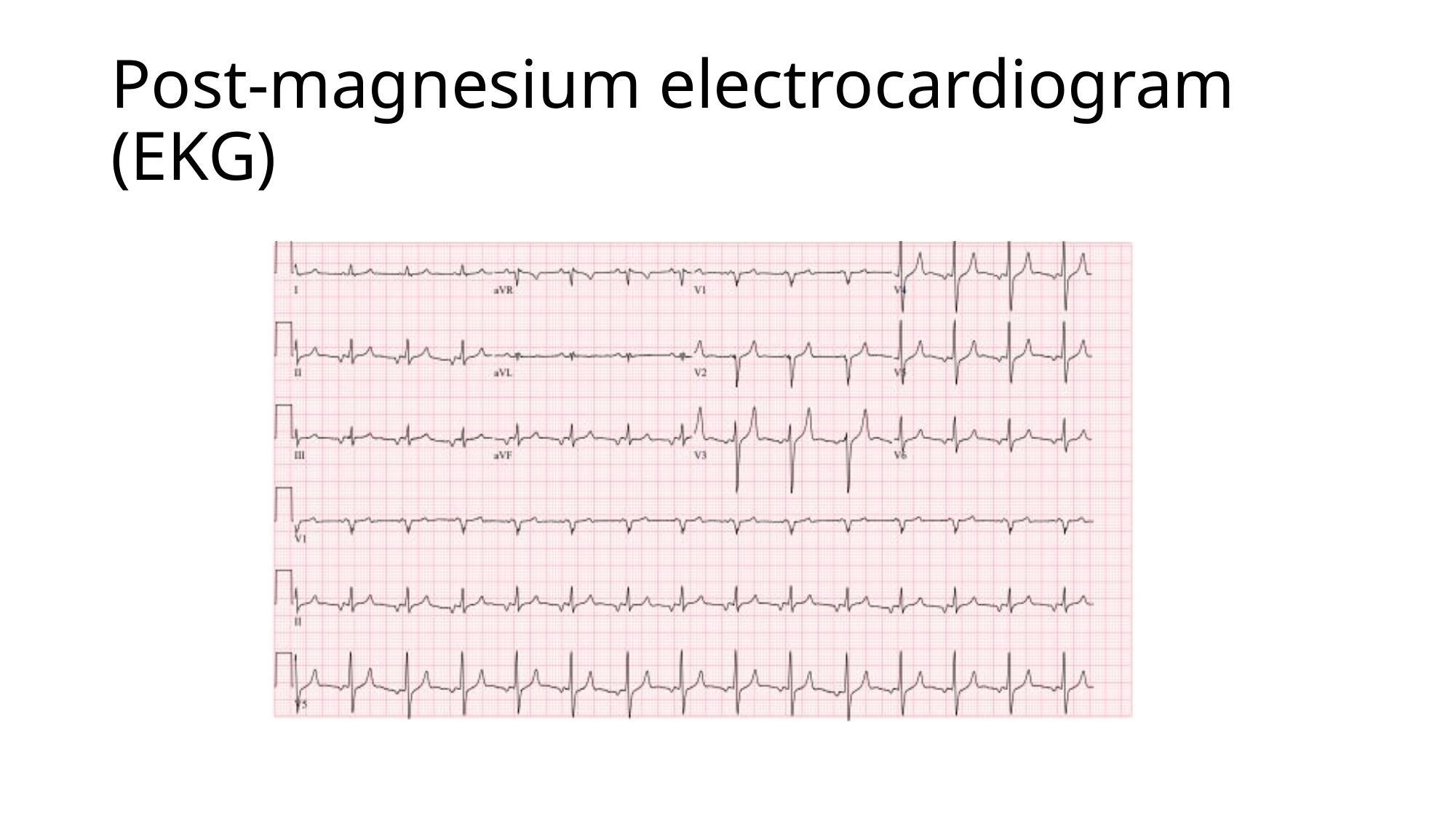

# Post-magnesium electrocardiogram (EKG)

## Slide 20
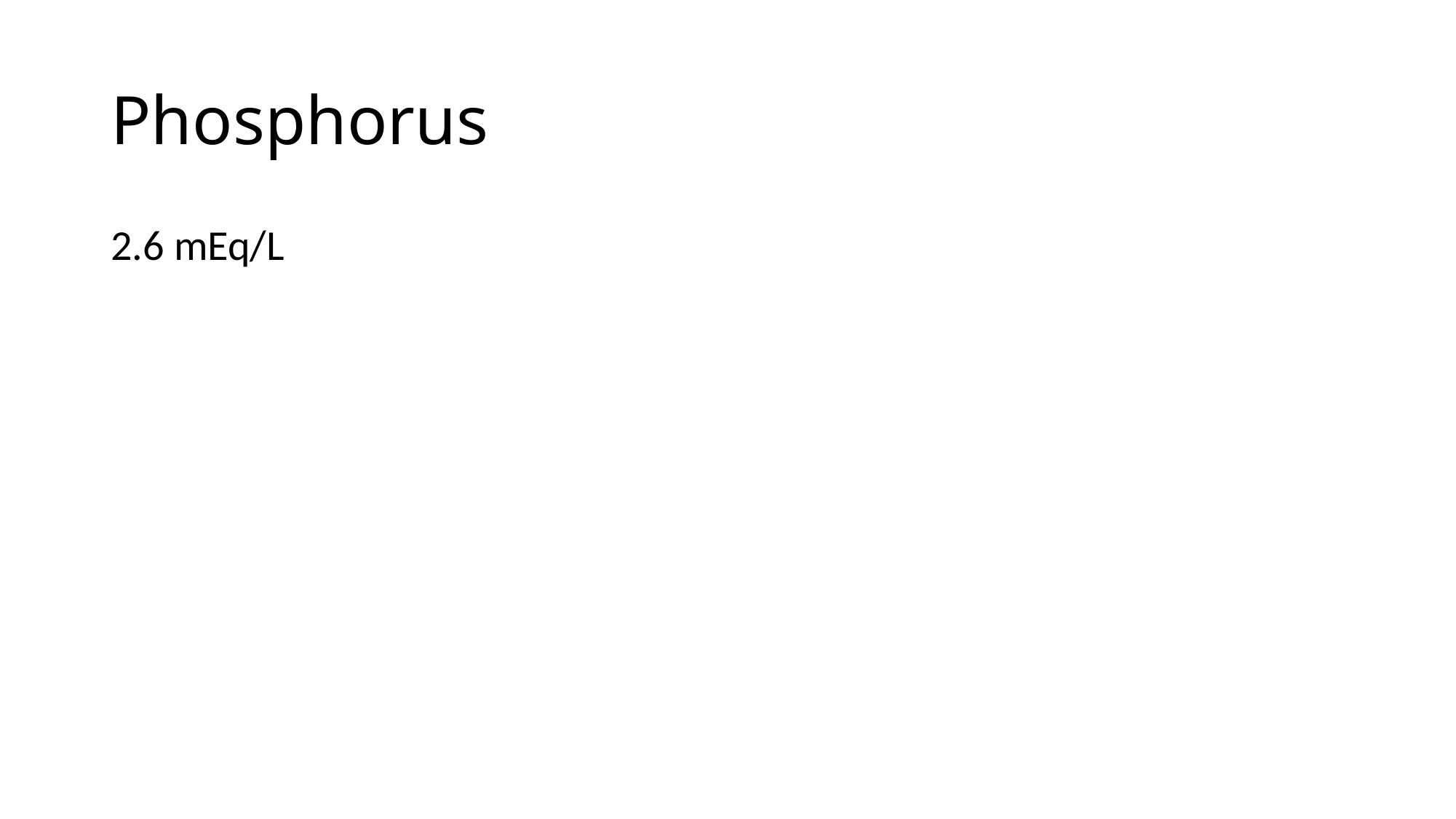

# Phosphorus
2.6 mEq/L
